# A *Plasmodium* Phospholipase Is Involved in Disruption of the Liver Stage Parasitophorous Vacuole Membrane

**DOI:** 10.1371/journal.ppat.1004760

**Published:** 2015-03-18

**Authors:** Paul-Christian Burda, Matthias A. Roelli, Marco Schaffner, Shahid M. Khan, Chris J. Janse, Volker T. Heussler

**Affiliations:** 1 Institute of Cell Biology, University of Bern, Bern, Switzerland; 2 Graduate School of Cellular Biology, University of Bern, Bern, Switzerland; 3 Leiden Malaria Research Group, Department of Parasitology, Leiden University Medical Center, Leiden, The Netherlands; MRC National Institute for Medical Research, UNITED KINGDOM

## Abstract

The coordinated exit of intracellular pathogens from host cells is a process critical to the success and spread of an infection. While phospholipases have been shown to play important roles in bacteria host cell egress and virulence, their role in the release of intracellular eukaryotic parasites is largely unknown. We examined a malaria parasite protein with phospholipase activity and found it to be involved in hepatocyte egress. In hepatocytes, *Plasmodium* parasites are surrounded by a parasitophorous vacuole membrane (PVM), which must be disrupted before parasites are released into the blood. However, on a molecular basis, little is known about how the PVM is ruptured. We show that *Plasmodium berghei* phospholipase, PbPL, localizes to the PVM in infected hepatocytes. We provide evidence that parasites lacking PbPL undergo completely normal liver stage development until merozoites are produced but have a defect in egress from host hepatocytes. To investigate this further, we established a live-cell imaging-based assay, which enabled us to study the temporal dynamics of PVM rupture on a quantitative basis. Using this assay we could show that PbPL-deficient parasites exhibit impaired PVM rupture, resulting in delayed parasite egress. A wild-type phenotype could be re-established by gene complementation, demonstrating the specificity of the PbPL deletion phenotype. In conclusion, we have identified for the first time a *Plasmodium* phospholipase that is important for PVM rupture and in turn for parasite exit from the infected hepatocyte and therefore established a key role of a parasite phospholipase in egress.

## Author Summary

Leaving their host cell is a crucial process for intracellular pathogens, allowing successful infection of other cells and thereby spreading of infection. *Plasmodium* parasites infect hepatocytes and red blood cells, and inside these cells they are contained within a vacuole like many other intracellular pathogens. Before parasites can infect other cells, the surrounding parasitophorous vacuole membrane (PVM) needs to be ruptured. However, little is known about this process on a molecular level and *Plasmodium* proteins mediating lysis of the PVM during parasite egress have not so far been identified. In this study, we characterize a *Plasmodium* phospholipase and show that it localizes to the PVM of parasites within hepatocytes. We demonstrate that parasites lacking this protein have a defect in rupture of the PVM and thereby in host cell egress. In conclusion, our study shows for the first time that a phospholipase plays a role in PVM disruption of an intracellular eukaryotic parasite.

## Introduction

The controlled exit of intracellular pathogens from host cells is an important step in infection and pathogenesis. This process is important for determining an organism’s life-cycle progression and the efficiency of a secondary infection and additionally the route and timing of egress may influence host immune responses [1]. Compared to what is known about the molecular mechanisms pathogens use to invade host cells, the process of host cell exit is much less understood. To escape from host cells, many pathogens have to disrupt two membranes, that of the vacuole they are contained within and the plasma membrane of the host cell. Although different molecules have been identified that play a role in the disruption of membranes, the precise mechanisms of membrane degradation are not well understood. For bacteria and intracellular protozoan parasites, pore-forming proteins (PFPs) have been shown to be involved in promoting vacuole escape. Similarly, bacterial phospholipases have also been identified as playing key roles in the disruption of vacuole membranes (reviewed in [1,2]).

*Plasmodium* parasites infect hepatocytes and red blood cells (RBCs) and inside these cells reside in a parasitophorous vacuole (PV). The PV membrane (PVM) is formed during invasion by invagination of the host cell plasma membrane [3] and is extensively modulated by the parasite through the insertion of *Plasmodium*-specific proteins and depletion of host proteins (reviewed in [4]). At the end of their development, parasites disrupt the PVM during the tightly regulated process of egress and are released, which is essential for progression of an infection.

For both blood and liver stage parasites, it has been shown that cysteine proteases play a key role in egress. In both stages, the general cysteine protease inhibitor E64 blocks egress from the PV and members of the *Plasmodium* serine repeat antigen (SERA) family are cleaved shortly before the release of parasites [5,6,7,8]. For the blood stage, subtilisin-like protease 1 (SUB1) and dipeptidyl peptidase 3 (DPAP3) have been identified to be part of a protease cascade resulting in parasite release [6,9] and recently it could be demonstrated that PbSUB1 also plays an important role for parasite egress at the end of liver stage development [10,11].

In addition to proteases, it has been demonstrated that perforin-like proteins and kinases are involved in parasite egress. *Plasmodium* parasites express a small, conserved family of proteins encoding perforin-like proteins (PPLPs) containing membrane-attack complex/perforin domains [12]. One of these proteins, PPLP1, has membranolytic activity and localizes to the PVM and RBC membranes of *Plasmodium falciparum* blood stages just before egress [13] and gametocyte stage parasites deficient in PPLP2 were unable to escape from their host RBC [14,15]. In addition, *Plasmodium* parasites deficient in either cGMP-dependent protein kinase (PKG) or the calcium-dependent protein kinase 5 (CDPK5) exhibit defects in parasite egress [16,17]. Furthermore, it has been shown that a liver stage-specific protein, LISP1, plays an important role in PVM disruption, but in contrast to the aforementioned proteins, LISP1 has no defined functional domain and its molecular function is unknown [18].

Despite this knowledge regarding the importance of proteases, kinases and perforin-like proteins for *Plasmodium* egress, the precise ordering of events and cellular mechanisms governing membrane degradation/disintegration remain unknown. Whether proteases have a direct role in membrane disruption by hydrolysis of membrane bound proteins or if they in turn activate other effectors is unclear.

Intracellular bacteria, such as *Listeria* and *Rickettsia*, use phospholipases to rupture vacuolar membranes [19,20] and it is reasonable to assume that other pathogens including *Plasmodium* parasites employ similar mechanisms to be liberated at the end of their development. However, the role of *Plasmodium* phospholipases in membrane disruption during egress has not been analyzed so far, although several putative phospholipases have been identified in *Plasmodium* based on sequence or structural similarity to phospholipases from other organisms (GeneDB.org).

Studies using phospholipase-C (PLC) inhibitors demonstrated that PLC activity is involved in multiple processes ranging from gametocyte development and sporozoite motility to egress of merozoites by regulating Ca^2+^ release [21,22,23]. Attempts to disrupt the gene encoding *Plasmodium berghei* PI-PLC (phosphoinositide-specific phospholipase C) have been unsuccessful, indicating an essential role for blood stage development [24]. Another protein on the surface of *P*. *berghei* sporozoites has been shown to exhibit phospholipase and membrane lytic activity *in vitro* [25]. This protein, *P*. *berghei* phospholipase (PbPL, PBANKA_112810), contains a predicted signal sequence and a carboxyl terminus that is 32% identical to the human lecithin:cholesterol acyltransferase, a secreted phospholipase [25]. Sporozoites deficient in PbPL expression have a reduced capacity to cross epithelial cell layers, indicating a role for PbPL in damaging host cell membranes.

In this study, we analyze the localization and role of PbPL during liver stage development. We show that PbPL is located at the PVM of liver stage parasites and that mutants deficient in PbPL exhibit a delayed egress as a result of impaired rupture of the PVM. Together, this is the first report of a protein with phospholipase activity that is involved in PVM disruption by a protozoan parasite.

## Results

### PbPL is expressed throughout liver stage development and localizes to the PVM

**Fig 1:**
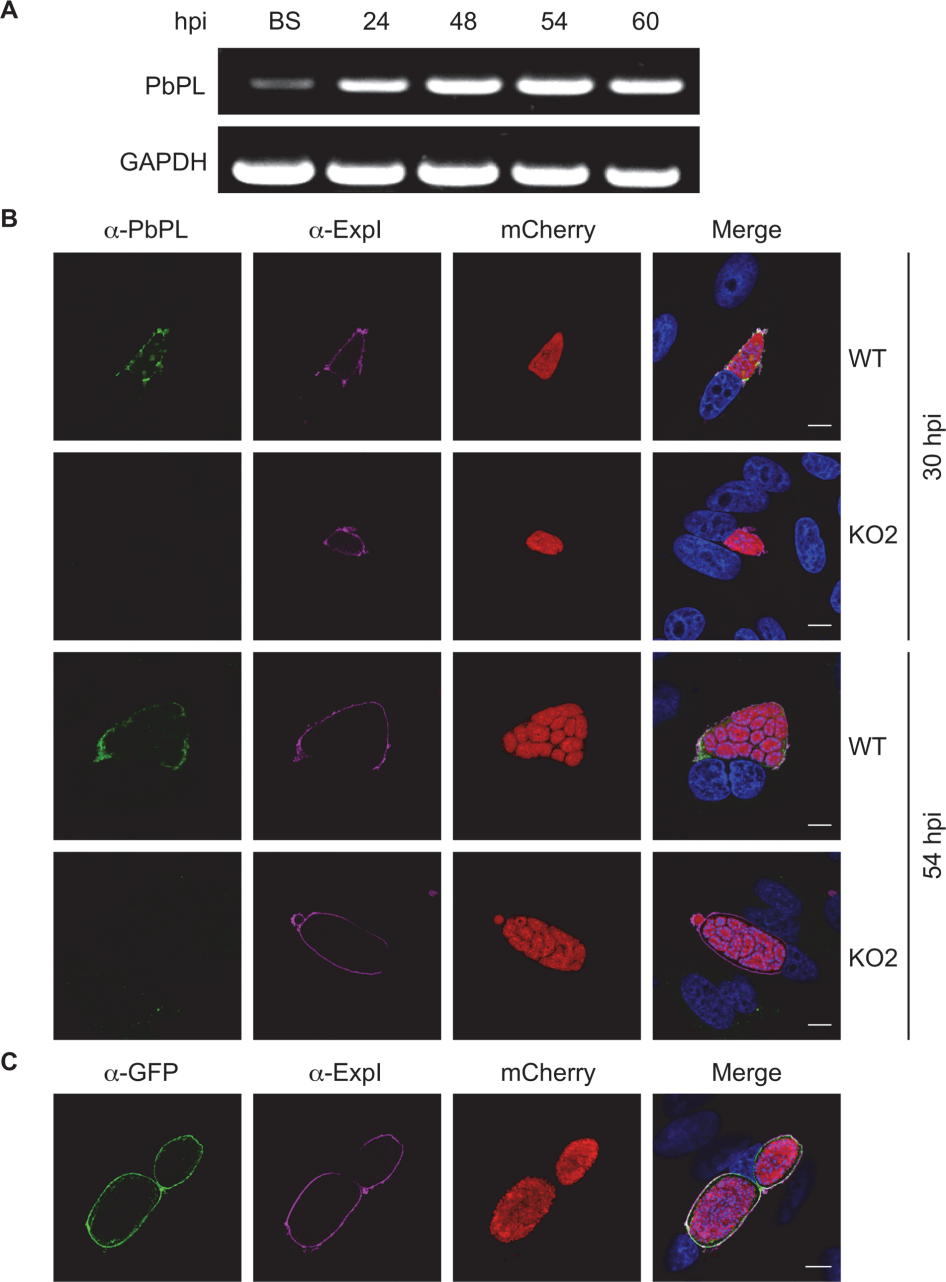
PbPL is expressed throughout liver stage development and localizes to the PVM. A) PbPL mRNA expression profile in blood stages (BS) and in liver stages at 24, 48, 54 and 60 hours post-infection (hpi). Transcripts were detected by RT-PCR, using primers specific for PbPL and *P*. *berghei* glyceraldehyde-3-phosphate dehydrogenase (GAPDH, PBANKA_132640) as a control. B) Endogenous PbPL localizes to the PVM. An antiserum against His-PbPL_195–312_ was raised in a mouse and used in IFA of wild-type (WT) and PbPL-knockout (KO2) liver stage parasites constitutively expressing cytosolic mCherry. Parasites were fixed at 30 and 54 hpi and additionally stained with an antiserum against the PVM marker protein ExpI. C) GFP-tagging of PbPL confirms PVM localization. PbPL was expressed as a GFP-fusion protein in *P*. *berghei* liver stage parasites additionally constitutively expressing cytosolic mCherry, which were fixed at 54 hpi and analyzed by IFA. PbPL (green), ExpI (purple), mCherry (red). The merged channels additionally contain DAPI-stained nuclei (blue). Scale bars = 10 μm.

PbPL was shown to be expressed on the surface of sporozoites and to have an important role during transmigration of sporozoites through cells [25]. However, it was not known whether PbPL is also expressed during liver stage development. We therefore first analyzed its transcription by RT-PCR, which showed mRNA expression of PbPL throughout liver stage development (Fig. 1A). To determine protein expression and PbPL localization, we generated a mouse antiserum against a hydrophilic fragment of PbPL (amino acid 195 to 312). Performing immunofluorescence assays (IFA) with the anti-PbPL antiserum revealed that PbPL colocalizes with the PVM resident protein exported protein I (ExpI, PBANKA_092670) in infected hepatocytes 30 and 54 hours post-infection (hpi). At 30 hpi, PbPL was also observed in vesicular structures within the parasite cytoplasm, which may be newly synthesized PbPL located in secretory vesicles being transported to the PVM. No signal was observed in liver stages of PbPL-knockout (KO) parasites (see below), confirming the specificity of the antiserum for PbPL (Fig. 1B). We further confirmed the localization by generating parasites expressing a PbPL-GFP fusion protein under the liver stage specific *lisp2* (PBANKA_100300) promoter [26], in which PbPL-GFP also localized to the PVM (Fig. 1C). These observations, that PbPL is expressed by parasites in infected hepatocytes and localizes to the PVM, may indicate that PbPL also plays a role in parasite development after sporozoite invasion.

### Generation of PbPL-KO parasites and PbPL complementation

**Fig 2:**
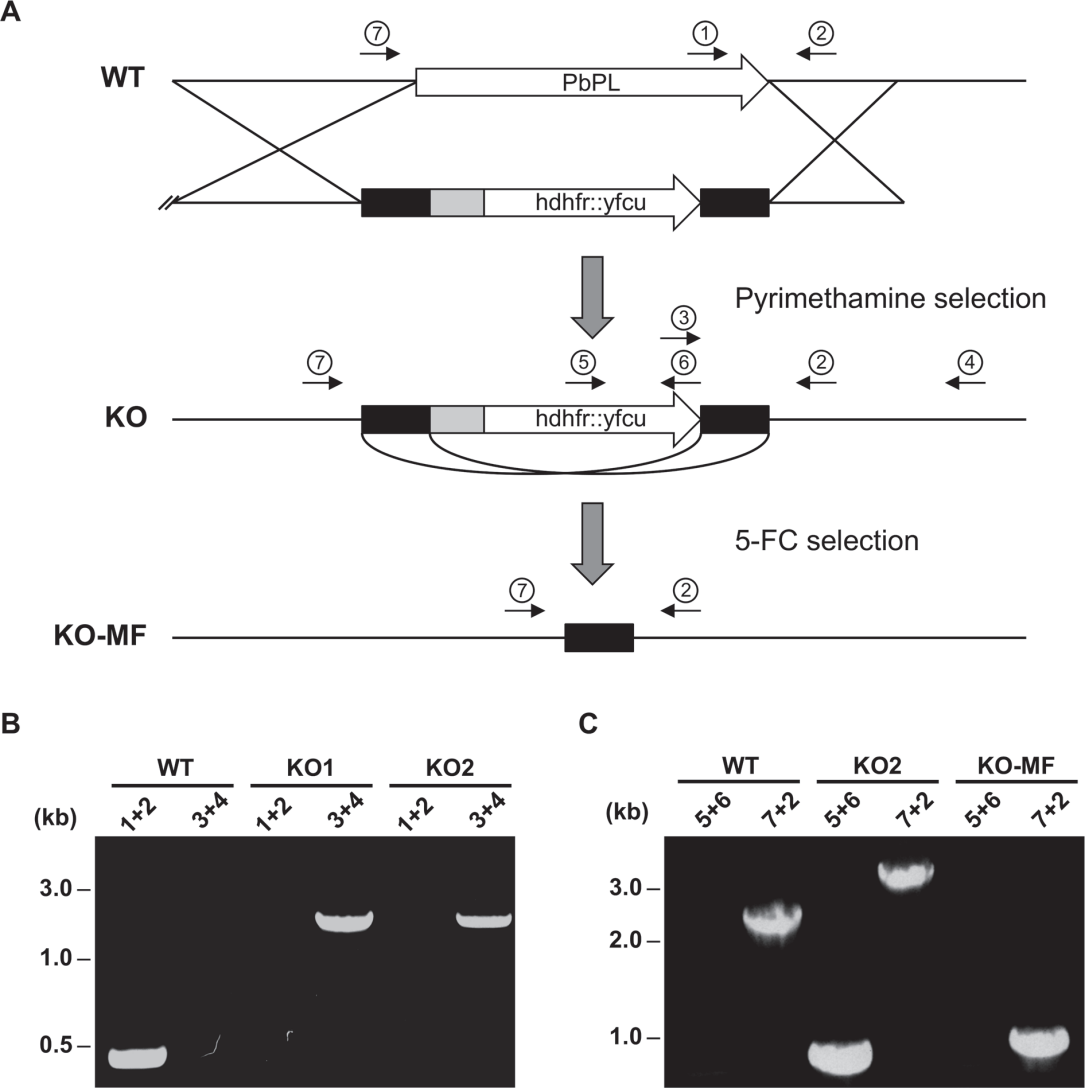
Generation and genotype analyses of PbPL-knockout parasite lines. A) Schematic representation of knockout (KO) strategy and marker recycling. Two clonal PbPL-KO parasite lines were generated by transfection of wild-type (WT) blood stage parasites with a plasmoGEM vector containing a fusion of the positive drug selectable marker *hdhfr* (human dihydrofolate reductase) and the negative marker *yfcu* (yeast cytosine deaminase and uridyl phosphoribosyl transferase) under the control of the *P*. *berghei eef1α* promoter (grey box) targeting the PbPL coding sequence by double crossover homologous recombination followed by pyrimethamine selection. The selection marker was removed by negative selection with 5-Fluorocytosine (5-FC), whereby marker-free PbPL-KO parasites (KO-MF) were selected that had undergone homologous recombination between the two *3’dhfr* untranslated regions (black boxes) present in the targeting vector flanking the *hdhfr*::*yfcu* cassette. Locations of primers used for PCR analysis are shown. B) Diagnostic PCR to confirm PbPL-KO clones. Primer 1 and 2 are expected to give a product of 398 bp in case of WT parasites, while primer 3 and 4 are expected to yield a product of 2058 bp for KO parasites. C) Diagnostic PCR to confirm successful removal of selectable marker. Primers 5 and 6 bind in the *yfcu* gene and are therefore expected to only give a product of 909 bp in case of selectable marker containing KO parasites. Primers 7 and 2 are expected to give a product of 2568 bp in case of WT, a product of 3782 bp for KO and a product of 1001 bp for KO-MF parasites. All primer sequences are listed in S1 Table.

To assess the role of PbPL in liver stage development, we sought to analyze a PbPL-KO parasite line. Unfortunately, the existing PbPL deletion mutant [25] shows insufficient fluorescence to allow liver stage development analysis using our assays. For this reason, we generated a new PbPL-KO parasite line by targeted deletion of the *pbpl* gene by double crossover homologous recombination (Fig. 2A). A gene-specific plasmoGEM vector [27,28] was transfected into blood stage schizonts of a marker-free *P*. *berghei* reporter line (mCherry_*hsp70*_) that expresses mCherry at high levels under the control of the *hsp70* (PBANKA_071190) regulatory sequences throughout the life cycle, making it particularly useful for fluorescence-based assays (S1 Fig.). Parasites of the mCherry_*hsp70*_ line are hereafter referred to as wild-type (WT) parasites. Successful deletion of PbPL was confirmed in two clonal KO parasite lines (KO1 and KO2) by diagnostic PCR (Fig. 2B).

**Fig 3:**
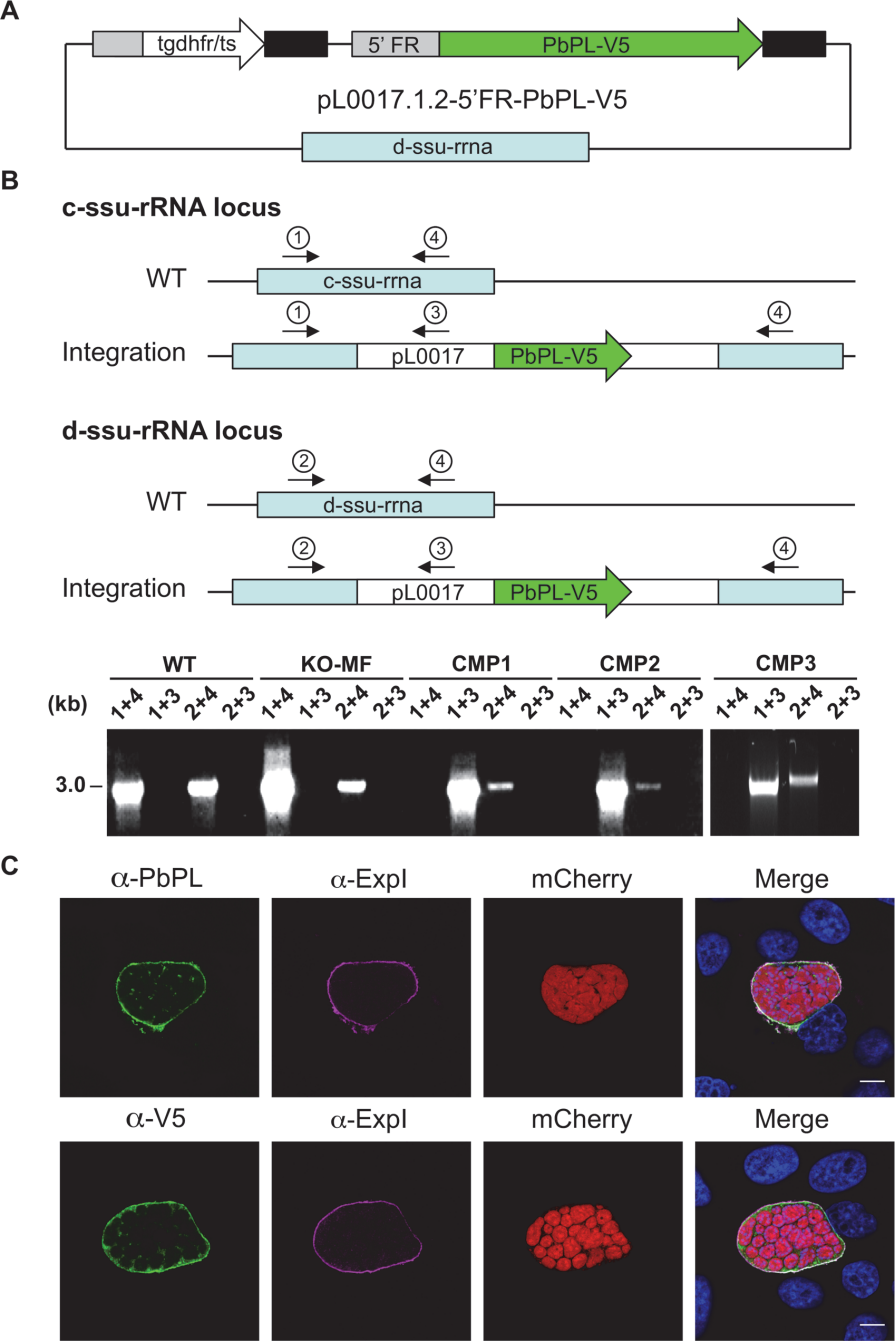
Generation and confirmation of complemented PbPL-knockout parasites. A) Schematic representation of the plasmid pL0017.1.2-5’FR-PbPL-V5 used to transfect marker-free PbPL-knockout (KO-MF) parasites, thereby generating complemented PbPL-KO (CMP) parasites. The PbPL coding sequence and the endogenous promoter region (1067 bp upstream of the start codon) were amplified from wild-type (WT) parasite gDNA by PCR and cloned in frame with a c-terminal V5-tag into a pL0017-derived plasmid. This vector integrates into the *c-* or the *d-ssu-rRNA* locus via single crossover recombination and conveys resistance to pyrimethamine. B) Diagnostic PCR of complemented parasite lines. Successful integration of the transfected plasmid into either of two possible loci in the *P*. *berghei* genome was tested by PCR. Locations of primers used for PCR analysis are shown. For each locus, one primer pair (1 and 4, 2 and 4, respectively) yields a PCR product of 3 kb if no integration has taken place. In case of successful integration, the primers are too far apart (>14 kb) to result in a complete PCR product under the chosen conditions. To further confirm integration, additional primer pairs (1 and 3, 2 and 3) were used, which only generate a PCR product of 3 kb if the plasmid has integrated. C) Complemented parasites express PbPL-V5 under the endogenous promoter. HepG2 cells were infected with complemented PbPL-KO sporozoites (CMP2) constitutively expressing cytosolic mCherry (red), fixed at 54 hpi and analyzed by IFA using an antiserum against PbPL (green, upper panel) or a monoclonal antibody against the V5-tag (green, lower panel) in combination with an antiserum against the PVM marker protein ExpI (purple). The merged channels additionally contain DAPI-stained nuclei (blue). Scale bars = 10 μm. IFAs are representative for the CMP1 and CMP3 parasite lines, which showed similar PbPL- and V5-stainings. All primer sequences are listed in S1 Table.

To validate that any potential mutant phenotype is the result of the absence of PbPL, we reintroduced the *pbpl* gene into KO parasites, taking advantage of the fact that the vector used for generation of the PbPL-KO contains a fusion of the positive drug selectable marker *hdhfr* (human dihydrofolate reductase) and the negative marker *yfcu* (yeast cytosine deaminase and uridyl phosphoribosyl transferase) under the control of the *P*. *berghei eef1α* promoter. To allow complementation, we first removed the selectable marker by treating KO2 parasites with 5-Fluorocytosine (5-FC), selecting for marker-free PbPL-KO parasites that had undergone homologous recombination between the two *3’dhfr* untranslated regions present in the targeting vector flanking the *hdhfr*::*yfcu* cassette (Fig. 2A). Successful removal of the selectable marker in a clonal KO parasite line was confirmed by diagnostic PCR (Fig. 2C). In a next step, we complemented these marker-free PbPL-KO parasites by transfection of a plasmid, in which expression of a V5-tagged PbPL is under the control of the endogenous *pbpl* promoter (Fig. 3A). We confirmed the correct complementation in three clonal lines (CMP1–3) by diagnostic PCR (Fig. 3B). In addition, PbPL expression during liver stage infection in these complemented lines was demonstrated by IFA using either our anti-PbPL antiserum or an anti-V5 antibody (Fig. 3C).

### PbPL-KO sporozoites have a defect in egress from oocysts

We first checked mosquito development of PbPL-KO parasites and found that they did not differ from WT and complemented parasites with respect to: (i) the kinetics of male gamete egress after *in vitro* gametocyte activation (S2A Fig.), (ii) formation of male exflagellation centers after *in vitro* activation (S2B Fig.), (iii) production of oocysts (S2C Fig.) and (iv) the production of sporozoites inside oocysts (midgut sporozoites, S2D Fig.). However, we detected fewer PbPL-KO sporozoites in both the mosquito hemolymph (S2E Fig.) and salivary glands (S2F Fig.) compared to both WT and complemented parasites. The reduced number of hemolymph and salivary gland sporozoites despite the production of normal numbers of sporozoites within oocysts indicates that PbPL-KO sporozoites have a defect in their egress from oocysts. A function of PbPL during sporozoite formation was not completely unexpected, since a previous study has shown that the *pbpl* promoter is active in oocysts [29].

### PbPL-KO parasites take longer to appear in the blood after sporozoite infection

**Fig 4:**
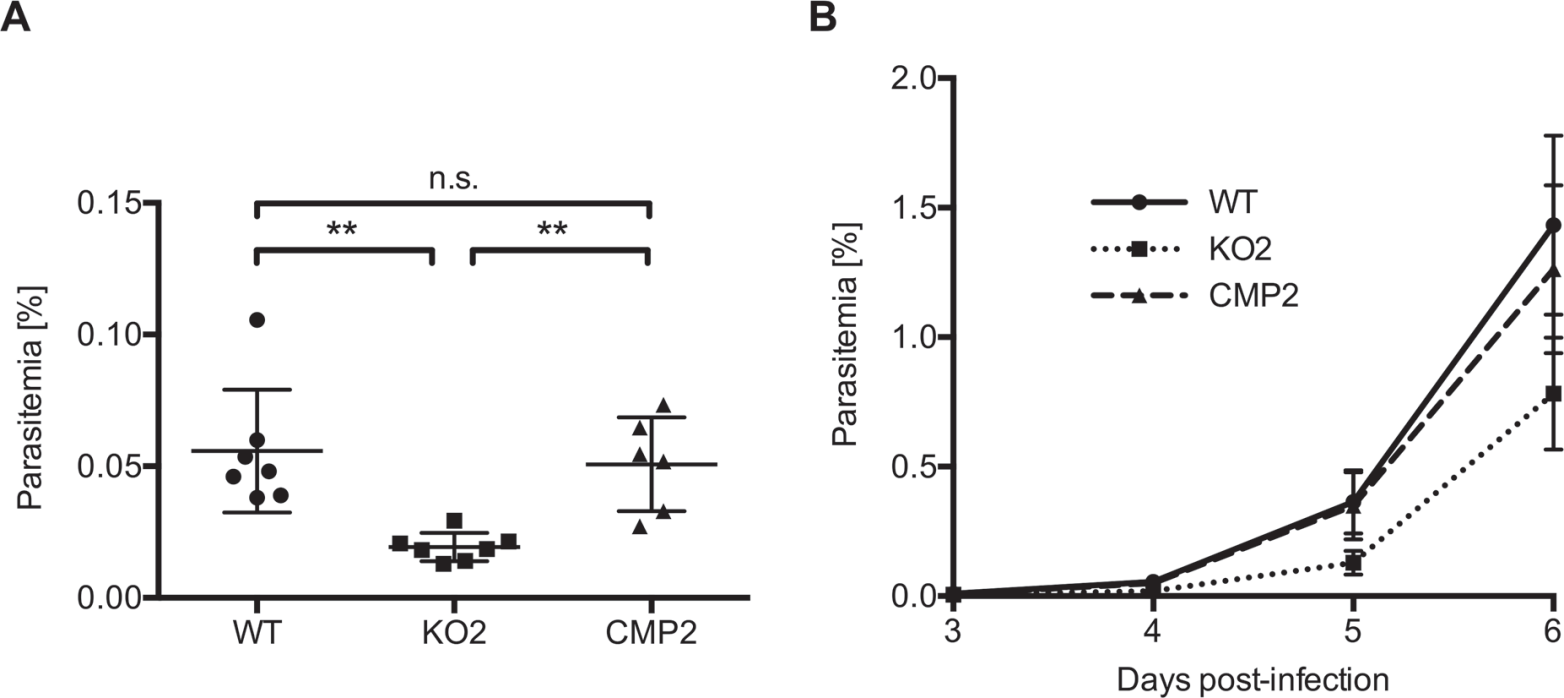
PbPL-knockout parasites take longer to appear in the blood after sporozoite infection. C57BL/6 mice were injected intravenously with 1,000 wild-type (WT), PbPL-knockout (KO2) or complemented PbPL-KO (CMP2) sporozoites and subsequent blood stage parasitemia was measured by FACS analysis. A) Blood stage parasitemia at day 4. B) Development of parasitemia between day 3 and 6 post-infection. Shown are means +/− SD of 6–7 infected mice per group. For statistical analysis of parasitemia at day 4, a one-way analysis of variance (ANOVA) followed by a Holm-Sidak multiple comparison test was performed (** p < 0.01, n.s. = not significant). See also S3 Fig.

When we infected C57BL/6 mice intravenously with 1,000 sporozoites of WT, KO or complemented parasites, the first blood stage parasites were detected in all mice 3 days after sporozoite injection. However, parasitemia for PbPL-KO parasites was significantly lower in comparison to both WT and complemented parasites on day 4 and subsequent days, as determined by FACS analysis (Fig. 4). These observations indicate either a reduced sporozoite infectivity, a defect during liver stage development or egress, or a reduced growth rate of blood stage parasites. To exclude that the lower parasitemia of PbPL-KO parasites after sporozoite infection is the result of a reduced growth rate of blood stages, we calculated the blood stage multiplication rate of PbPL-KO parasites from the increase in parasitemia after sporozoite infection (S3A Fig.). In addition, we analyzed blood stage growth in mice after intravenous injection of 1,000 WT, PbPL-KO or complemented blood stage parasites (S3B Fig.). The results of these analyses showed that the blood stage growth of PbPL-KO parasites was comparable to that of WT and complemented parasites, indicating that PbPL does not play a critical role during blood stage development. To analyze whether the reduced parasitemia is the result of a reduced infection of liver cells by sporozoites, we quantified the liver load of mice infected with the same number of WT, PbPL-KO or complemented sporozoites 38 hpi by real-time PCR. In agreement with previous findings [25], we were not able to detect a difference between the parasite lines (S3C Fig.), indicating a normal infectivity of PbPL-KO sporozoites.

In conclusion, the reduced parasitemia of PbPL-KO parasites after sporozoite infection in combination with comparable liver loads and the absence of an obvious blood stage phenotype indicates that PbPL-KO parasites either take longer to emerge from the liver or that fewer infectious merozoites are released.

### PbPL-KO parasites undergo normal liver stage development but show impaired merozoite release

**Fig 5:**
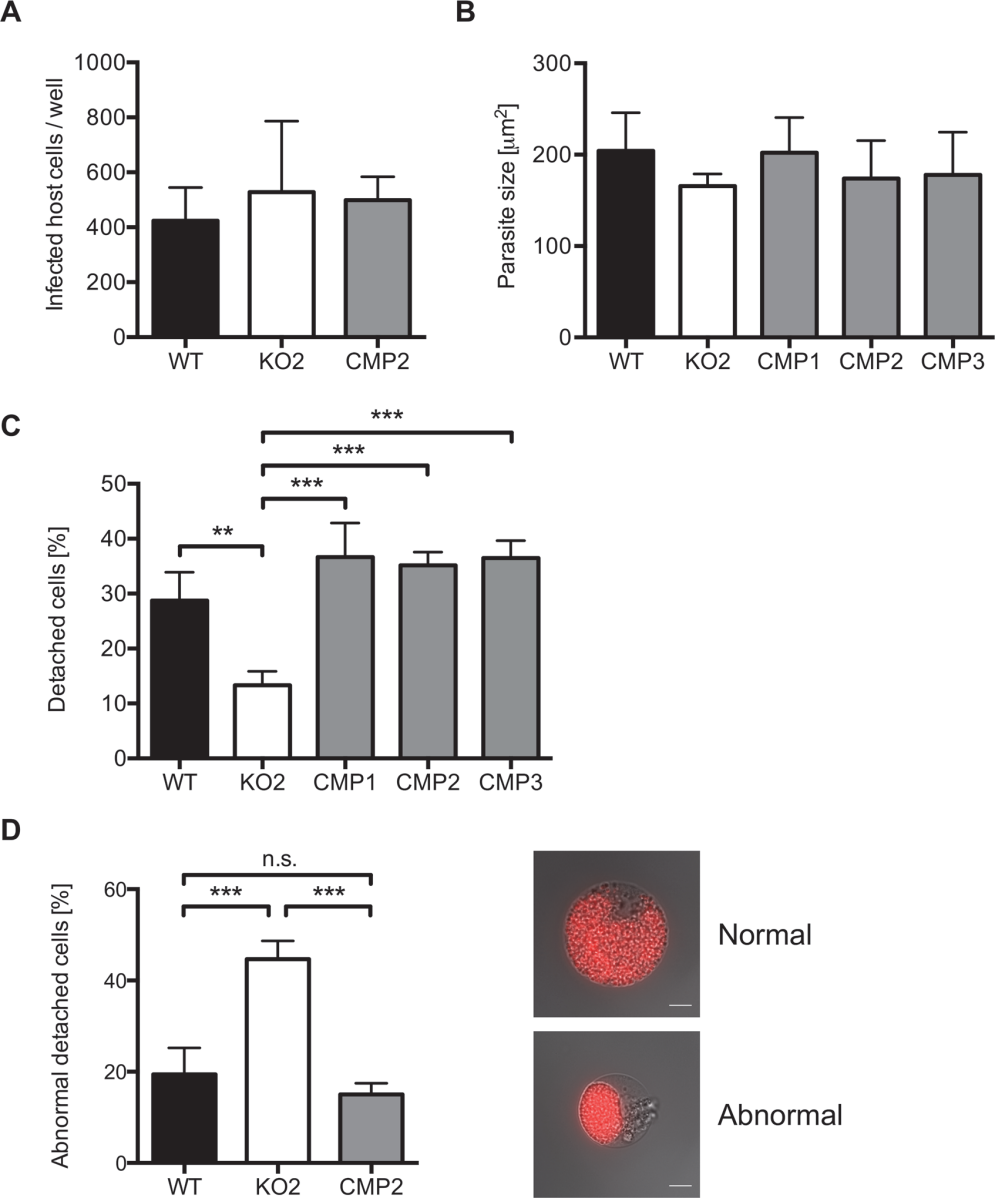
PbPL does not affect liver stage growth but plays a role in detached cell formation A) PbPL-knockout (KO2) sporozoites have a similar infectivity as wild-type (WT), and complemented PbPL-KO (CMP2) sporozoites. For determination of sporozoite infectivity, HepG2 cells were infected with 10,000 WT, KO2 or CMP2 sporozoites and the average number of infected host cells per well was quantified 48 hpi in triplicate. Numbers of infected host cells were not statistically different from each other (one-way ANOVA, p = 0.6892). B) PbPL-knockout parasites grow normally in size. HepG2 cells were infected with WT, KO2 and complemented PbPL-KO (CMP1–3) sporozoites. 48 hpi, parasite size (area) was determined by density slicing using ImageJ. For each parasite line, the average size of 50–100 parasites was determined in each of three separate experiments. Parasites did not show a significant difference in size (one-way ANOVA, p = 0.6567). C) PbPL-KO parasites produce fewer detached cells (DCs). DCs in the supernatant were counted at 65 hpi in triplicate and were normalized to the number of infected cells at 48 hpi. D) Detached cells from PbPL-KO parasites show an abnormal morphology. DCs were harvested at 65 hpi and the percentage of cells with an abnormal morphology was determined. DCs with abnormal morphology were defined by merozoites still being clustered in the PV in contrast to merozoites freely distributed in the host cell in DCs with normal morphology. A representative image of DCs with normal and abnormal morphology is shown. Scale bars = 10 μm. For all experiments means +/− SD of three to four independent experiments are shown. For statistical analysis a one-way ANOVA followed by a Holm-Sidak multiple comparison test was performed (** p < 0.01, *** p < 0.001, n.s. = not significant). See also S4 Fig.

To better characterize the PbPL-KO liver stage phenotype, we next analyzed in detail the intrahepatic development of PbPL-KO parasites *in vitro*. To again exclude differences in sporozoite infectivity, we infected HepG2 cells with the same number of WT, KO and complemented sporozoites and counted infected hepatocytes at 48 hpi. KO sporozoites showed a similar infectivity to HepG2 cells in comparison to WT and complemented sporozoites (Fig. 5A), further supporting our *in vivo* analyses.

To investigate whether PbPL plays a role during the growth of *Plasmodium* liver stages, we measured the size of intrahepatic parasites *in vitro* at 48 hpi. No significant differences in size were observed between WT, KO and complemented parasites, suggesting that PbPL is not involved in liver stage development prior to this stage (Figs. 5B and S4A).

We subsequently investigated a potential role of PbPL during the final stages of liver stage schizogony, merozoite formation and egress from host hepatocytes. First, we showed by IFA that expression and localization of the merozoite surface protein 1 (MSP1, PBANKA_083100) and the PVM marker Exp1 in intrahepatic PbPL-KO parasites was the same as in WT parasites (S4B Fig.).

Next, we counted the number of detached cells produced at the end of intrahepatic development, as a marker for the final phase of liver stage development *in vitro* [5]. Cells detach upon formation of merozoites and rupture of the PVM, followed by the release of merozoites into the hepatocyte cytoplasm, which typically occurs between 55 and 60 hpi [5]. We observed that PbPL-KO parasites produced approximately 60% fewer detached cells compared to WT parasites (Figs. 5C and S4C). Furthermore, a significant proportion of detached cells in the KO population showed an aberrant morphology; merozoites were not released into the hepatocyte cytoplasm but remained clustered together (Fig. 5D). In complemented parasites, the WT phenotype of detached cells was completely rescued (Fig. 5C, D).

**Fig 6:**
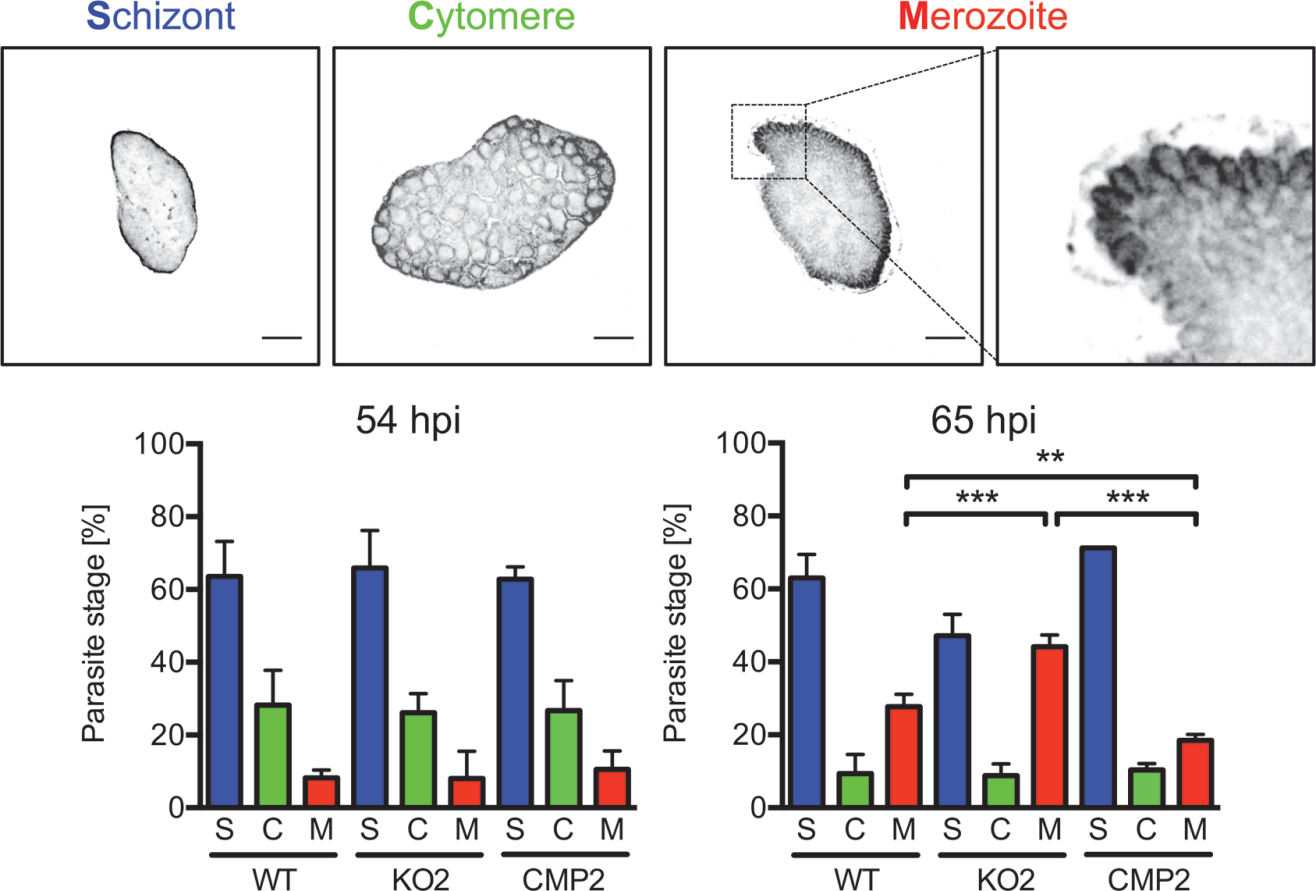
PbPL is involved in merozoite release. HepG2 cells were infected with wild-type (WT), PbPL-knockout (KO2) and complemented PbPL-KO (CMP2) sporozoites. The percentage of attached hepatocytes containing schizont (S), cytomere (C) and merozoite (M) stage parasites was determined at 54 and 65 hpi. Schizont stages are either negative for the merozoite surface protein MSP1 or display an MSP1 staining only at the parasite plasma membrane without invaginations. Cytomere stages are defined by their MSP1-positive parasite plasma membrane with clear invaginations, while in merozoite-containing hepatocytes, individual merozoites are surrounded by MSP1 staining. Representative MSP1 staining of each parasite stage is shown at the top. For each time point, 50–100 parasites were analyzed. Scale bars = 10 μm. Shown are means +/− SD of three independent experiments. For statistical analysis a one-way ANOVA followed by a Holm-Sidak multiple comparison test was performed (** p < 0.01, *** p < 0.001).

The reduced detachment in the case of mutant parasites could result from fewer parasites that successfully form merozoites or from a defect in PVM disruption. In the latter case, an increase in attached hepatocytes containing merozoites may be predicted because daughter cells would be produced normally but due to a defect in PVM disruption, cell detachment would not occur. To distinguish between these possibilities, we quantified attached hepatocytes containing schizont, cytomere and merozoite stages at 54 and 65 hpi. At 54 hpi, before PVM rupture, no significant difference in the number of hepatocytes containing merozoites existed between WT, PbPL-KO and complemented parasites, indicating normal merozoite formation up to this time point (Fig. 6). In contrast, at 65 hpi, a time point where the PVM is disrupted in normally developed WT parasites, a significantly higher number of attached hepatocytes containing merozoites was seen for PbPL-KO parasites compared to WT parasites, indicating impaired merozoite release and a potential defect in PVM disruption (Fig. 6).

We were able to rescue the WT phenotype by complementation, although complemented parasites showed a small but statistically significant increase in merozoite release compared to WT parasites in this assay. This difference in merozoite release could be the result of slightly increased PbPL-expression levels in complemented parasites compared to WT parasites. This may be explained by the process of complementation, where the DNA construct encoding the *pbpl* gene was introduced into the PbPL-KO genome by single-crossover recombination, possibly resulting in multiple insertions of the DNA construct. This increase in *pbpl* gene copy number could then lead to higher PbPL expression levels compared to that in WT parasites. Further, even the expression level of a single introduced PbPL expression construct might result in differences in PbPL expression, since we used only 1067 bp upstream of the *pbpl* start codon as a promoter region in the complementation vector, and consequently may not have captured the entire *pbpl* promoter, resulting in altered expression levels.

### PbPL-KO parasites exhibit a defect in PVM disruption

**Fig 7:**
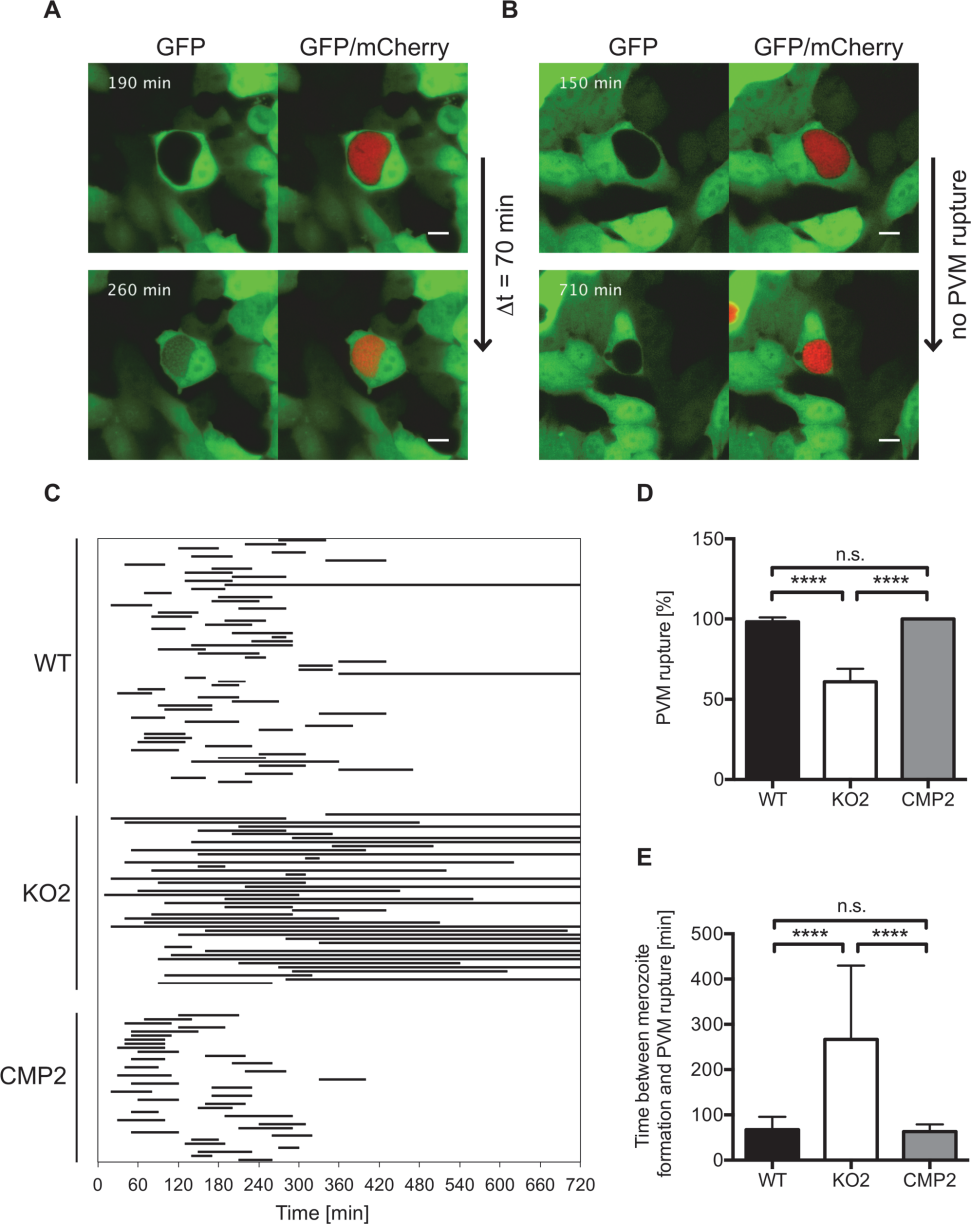
PbPL mediates disruption of the PVM. HepG2 cells expressing GFP (green) were infected with mCherry-expressing wild-type (WT), PbPL-knockout (KO2) and complemented PbPL-KO (CMP2) sporozoites (red). The percentage of merozoite-forming parasites that ruptured the PVM and the time difference between successful formation of merozoites and PVM rupture was measured by quantitative live-cell imaging. The influx of GFP into the PV was used as a measure of PVM rupture. Imaging was started around 55 hpi and lasted for 12 hours. Representative images for WT (A) and PbPL-KO (B) parasites are shown. The upper images show the time point of successful merozoite formation, at which individual merozoites were visible and all larger yet undivided parts of the parasite cytoplasm, typical of the cytomere stage, had disappeared. The lower images show the time point of PVM rupture (GFP influx) in a host cell infected with a WT parasite and the end point of imaging of a host cell infected with a PbPL-KO parasite, in which the PVM did not rupture (the course of events are better visible in the corresponding S1–S3 Movies). Scale bars = 10 μm. C) Time between formation of merozoites and PVM rupture. Each line represents the time difference between successful merozoite formation (beginning of line) and PVM rupture (end of line), as illustrated in A and B, and corresponds to one analyzed parasite. Continuous lines indicate parasites that did not rupture the PVM at all, which were not considered for determination of the average PVM rupture time in E. D) Percentage of merozoite-forming parasites that ruptured the PVM. The percentage of PVM rupture was determined in 3 (KO2, CMP2) or 6 (WT) imaging sessions, in which the number of parasites that successfully developed to merozoites within the first 6 hours of imaging was set to 100% in each experiment. Based on these, the percentage of parasites that successfully ruptured the PVM was calculated. E) Elapsed time from merozoite formation to PVM rupture. In D and E, means +/− SD are shown. Data were acquired in and are representative of 3 (KO2, CMP2) or 6 (WT) imaging experiments, in which a total of 61 WT, 43 KO2 and 37 CMP2 parasites were analyzed. For statistical analysis a one-way ANOVA followed by a Holm-Sidak multiple comparison test was performed (**** p < 0.0001, n.s. = not significant). See also S1–S3 Movies.

To further analyze impaired merozoite release in PbPL-KO parasites and a potential role of PbPL in PVM disruption, we infected GFP-expressing HepG2 cells with WT and PbPL-KO parasites and analyzed their intrahepatic development from the cytomere stage to cell detachment by live-cell time-lapse microscopy. An intact PVM is impermeable to host cell-derived GFP, whereas PVM rupture leads to a rapid GFP influx into the PV [30]. Analysis of GFP influx by live-cell time-lapse microscopy therefore allows the determination of the percentage of merozoite-forming parasites with a disrupted PVM and quantification of the speed of PVM disintegration. Nearly all WT parasites that developed to the merozoite stage were able to disrupt the PVM, with an average time of 70 minutes (Fig. 7, S1 Movie). In contrast, PbPL-KO parasites that developed to the merozoite stage either did not rupture the PVM at all or this process was significantly delayed (Fig. 7, S2 Movie). Importantly, the WT phenotype was completely rescued in complemented parasites (Fig. 7, S3 Movie). In conclusion this experiment shows that in the absence of PbPL, PVM rupture is compromised, confirming the results of the detached cell (Figs. 5C, and S4C) and the stage quantification assays (Fig. 6) and providing a perfect explanation for the observed delay in development of blood stage parasitemia after sporozoite infection (Fig. 4).

## Discussion

In intracellular bacterial pathogens, phospholipases have been shown to play a key role in host cell exit [1,2], whereas the role of this class of proteins in intracellular protozoans, including *Plasmodium* parasites, was unknown. In this study we have identified PbPL, a *Plasmodium* protein with phospholipase activity, as having a key role in disruption of the liver stage PVM. This is the first time that a *Plasmodium* phospholipase has been implicated in egress from a host cell.

Bhanot *et al*. have previously found that PbPL has phospholipase activity, is expressed on the surface of sporozoites and that it has a role in damaging host cell membranes, thereby assisting in the migration of sporozoites to the hepatocyte [25]. Together with our observations, demonstrating that PbPL-KO parasites are impaired in their egress from oocysts and from hepatocytes, this protein appears to have important functions in three life cycle stages prior to the blood stage of infection:

(i) in oocysts, where it plays a role in egress of sporozoites, (ii) in sporozoites, where it plays a role in migration through host tissue mediating hepatocyte invasion and (iii) in liver stages, where it is involved in PVM rupture mediating efficient merozoite release.

When Bhanot *et al*. injected a large number of PbPL-deficient sporozoites intravenously, they did not observe a prolonged prepatent period by blood smear. However, they observed a delayed prepatency when a much lower number of sporozoites were transferred by mosquito bite [25]. Our own observations principally confirm the finding of Bhanot *et al*., as the first blood stage parasites were detected in all mice 3 days after sporozoite injection. However, determination of parasitemia by the more sensitive method of FACS analysis revealed a significantly lower parasitemia for KO parasites, suggesting that PbPL also contributes to parasite egress *in vivo*.

In support of the function PbPL has in PVM disruption, the protein is located at the PVM, already being detectable there at 30 hpi, some time before the actual rupture of the PVM occurs. This may suggest that in the developing liver stage parasite, PbPL remains in an inactive state and its activation results in PVM rupture. A coordinated cascade of events involving kinases and resulting in the activation of proteases has been defined as being important for parasite release (reviewed in [31]) and this signaling and protease activation cascade may also include PbPL activation, for example by phosphorylation or proteolytic cleavage. As kinases and proteases are not likely membranolytic, an attractive scenario is that their activation converges on lipases like PbPL. In line with this hypothesis, proteolytic activation of phospholipases was already shown for a *Listeria* PLC [32] and for several secreted phospholipases of *Staphylococcus* (reviewed in [33]). Candidates for a potential proteolytic activation of PbPL could be the proteases of the SERA family that, like PbPL, also localize to the PVM in infected hepatocytes [7,8]. However, so far proteolytic cleavage of substrates has not been directly shown for any of the *P*. *falciparum* or *P*. *berghei* SERAs. Direct and indirect evidence that SERAs with a cysteine residue in the active center are indeed real proteases came from two recent studies: One study showed that the putative active site cysteine of *P*. *falciparum* SERA6 is essential and that the *P*. *berghei* orthologue of SERA6 could be converted by SUB1-mediated cleavage to an active cysteine protease showing autoprocessing activity [34]. The other study revealed that the exchange of the serine residue in the active center of *P*. *falciparum* SERA5 to a cysteine allows peptide binding and cleavage [35]. These observations, the localization of different SERAs in the PV or even in the PVM and the fact that expression of the majority of *P*. *berghei* SERAs is restricted to the last few hours before merozoite egress from infected hepatocytes [7,8], suggest a possible role in initiation of PVM rupture by, for example, processing and activation of PbPL or other membranolytic enzymes.

While our study demonstrates that PbPL is involved in disruption of the PVM, our observations also show that a proportion of PbPL-KO parasites are able to disrupt the PVM inside the infected hepatocyte in the absence of PbPL. This indicates that PVM disruption can be brought about by other parasite molecules, albeit less efficiently, or can occur by non-specific (possibly mechanical) processes. The ability of the parasite to utilize multiple exit strategies is further supported by the absence of any obvious blood stage phenotype in PbPL-deficient parasites. Interestingly, the absence of other *Plasmodium* proteins shown to be involved in egress, such as LISP1 [18] and the perforin-like molecule PPLP2 [14,15], also did not result in an absolute ‘non-egress’ phenotype. Host cell egress is such a critical process for intracellular parasites that presumably several different effector proteins and mechanisms exist, which may have partially overlapping, redundant or even synergistic functions. Therefore, the absence of just one effector molecule, such as PbPL, may only lead to a partial defect in egress. An example of this functional redundancy has been described in *Listeria* [19], where separate deletion of two phospholipases had only moderate effects on the infectivity to mice (2–20 fold reduction), whereas the deletion of both phospholipases together severely impaired infectivity (500 fold reduction). In line with this, the apparently moderate phenotype resulting from the deletion of PbPL enables the generation of double mutants in the future, providing an opportunity to identify other proteins working in conjunction with PbPL, and thereby may help us to more completely understand the process and the hierarchy of events that facilitate parasite exit from the host cell.

Our current working hypothesis is that PbPL works together with other phospholipases or pore-forming proteins (PFPs), like the already mentioned *Plasmodium* perforin-like proteins [12]. In general, the combination of membranolytic enzymes might be specific for each egress event in the life cycle and for different *Plasmodium* species, depending on the composition of the PVM that the parasite has to rupture and the exit strategy employed. It makes sense, for example, that the lysis of the RBC PVM differs from that of infected hepatocytes, as in RBCs the same enzymes probably also rupture the host plasma membrane within seconds, whereas the plasma membrane of hepatocytes needs to be preserved for several hours until merosomes are formed and reach the blood stream [5].

We consider PFPs as promising candidates to be involved in PbPL-mediated PVM disruption, as they were shown to play a key role in host cell egress of intracellular bacteria, for which it has been suggested that PFPs and phospholipases may act in concert [36]. Although the detailed molecular basis of this is unknown, PFPs might make certain membrane leaflets accessible for the action of lipases. In *Plasmodium* the PFPs of the perforin-like protein family consist of five conserved proteins all containing a membrane-attack complex/perforin domain [12]. Members of this family have been shown to be important for ookinete and sporozoite host cell traversal [37,38,39] and have also been implicated in host cell egress by asexual and sexual blood stage parasites [13,14,15]. However, studies on the role of perforin-like proteins at the end of liver stage development are so far missing and it can only be speculated that these proteins contribute to parasite egress from infected hepatocytes. Interestingly, key roles of PFPs in egress have also been established for several other protozoan parasites, including *Trypanosoma cruzii*, *Leishmania* and *Toxoplasma gondii* [40,41,42], but a putative function of phospholipases in exit of these parasites from their host cells has not been investigated so far.

Taken together, our study identifies PbPL as the first *Plasmodium* phospholipase that is important for PVM disruption and in turn for parasite exit from the infected hepatocyte and therefore establishes a key role of a parasite phospholipase in egress. We strongly believe that PbPL, because its function is significant but not lethal, offers the unique opportunity to learn more about parasite egress strategies. We now aim to identify the specific combination of membranolytic enzymes needed for membrane rupture and to understand the mechanisms by which these enzymes act together, as they might represent a new class of parasite-specific targets for intervention.

## Materials and Methods

### Ethics statement

All experiments performed at the University of Bern were conducted in strict accordance with the guidelines of the Swiss Tierschutzgesetz (TSchG; Animal Rights Laws) and approved by the ethical committee of the University of Bern (Permit Number: BE109/13). All experiments performed at the LUMC were approved by the Animal Experiments Committee of the Leiden University Medical Center (Permit Number: DEC 12042). The Dutch Experiments on Animals Act was established under European guidelines (EU directive no. 86/609/EEC regarding the Protection of Animals used for Experimental and Other Scientific Purposes).

### Experimental animals

Mice used in the experiments were between 6 and 10 weeks of age and were from Harlan Laboratories, Charles River or bred in the central animal facility of the University of Bern. Mosquito feeds were performed on mice anaesthetized with Ketavet/Domitor and all efforts were made to minimize suffering.

For the generation of mCherry_*hsp70*_ parasites, Swiss mice were used. The *in vivo* phenotype of PbPL-KO parasites was analyzed in C57BL/6 mice, while for all other experiments Balb/c mice were used.

### Mosquito infection

Infections of mice were initiated by intraperitoneal injection of *P*. *berghei* blood stabilates. When these mice had a parasitemia of 4%, 150 μl or 40 μl of infected blood were injected intraperitoneally or intravenously, respectively, into mice that had received an intraperitoneal injection of 200 μl phenylhydrazine (6 mg/ml in PBS) 2–3 days before. At day 3 or 4 after infection, mice with a parasitemia of at least 7% were anaesthetized for 1 hour to allow feeding of 150 female *Anopheles stephensi* mosquitoes. The next day, unfed mosquitoes were removed. Mosquitoes were kept at 20.5°C with 80% humidity and for infection experiments, sporozoites were isolated from infected salivary glands 16–27 days after the infective blood meal.

### Culture and infection of HepG2 cells

HepG2 cells (obtained from the European cell culture collection) were cultured as described before [30]. For infection, either 3 or 5 x 10^4^ cells were seeded in 24-well plates with or without coverslips. The next day, *P*. *berghei* sporozoites were isolated from the salivary glands of infected *A*. *stephensi* mosquitoes and added to HepG2 cells in culture medium additionally containing 2.5 μg/ml amphotericin B (PAA Laboratories). After an incubation period of 2 hours, the sporozoite-containing medium was removed and fresh infection medium was added. Subsequently, medium was changed once per day.

### Gene expression analysis

Total RNA was isolated from 0.05% saponin-treated *P*. *berghei* infected red blood cells and infected HepG2 cells 24, 48, 54 and 60 hpi using the NucleoSpin RNA II kit (Macherey-Nagel). Random-primed cDNA synthesis was performed using GoScript reverse transcriptase (Promega) and the resulting cDNA was then used as a template in PCR reactions using GoTaq Flexi DNA polymerase (Promega) with the primer pairs PbPL-expression-fw/PbPL-expression-rev and GAPDH-expression-fw/GAPDH-expression-rev. All primer sequences are listed in S1 Table.

### Cloning of DNA constructs

All PCR reactions were performed using Phusion DNA polymerase (NEB). PCR products were routinely cloned into pJET1.2 (Fermentas) and confirmed by sequencing.

For generation of the PbPL bacterial expression vector parallel-1-His-PbPL_195–312_, the coding sequence corresponding to a hydrophilic fragment of PbPL ranging from amino acid 195 to 312 was amplified from blood stage cDNA using primer pair PbPL-antiserum-fw/PbPL-antiserum-rev, which was then cloned into the parallel-1-His vector [43] using *Bam*HI and *Xho*I restriction sites.

The PbPL-GFP expression vector pL0043^LS^PbPL-GFP^C^mCherry was generated by first amplifying the PbPL coding sequence from blood stage cDNA using primer pair PbPL-GFP-fw/PbPL-GFP-rev, which was subsequently digested with *Bgl*II and ligated into the *Bam*HI digested liver stage-specific expression vector pGFP_103464_ [26] in frame with GFP. From there the ^LS^PbPL-GFP expression cassette was cloned via *Eco*RV and *Kpn*I into the pL0043 vector [44], which targets the *P*. *berghei 230p* locus by double crossover homologous recombination. Finally, a constitutive mCherry expression cassette was integrated via *Kpn*I, which had been amplified before from the p^C^mCherry plasmid [45] using primers mCherry-fw and mCherry-rev.

For generation of the PbPL-complementation vector pL0017.1.2-5’FR-PbPL-V5, the vector pL0017.1.2 was generated at first. For this, the GFP coding sequence was excised from pGFP_103464_ [26] using *Bam*HI and *Xba*I digestion and then replaced by a double-stranded DNA oligonucleotide (obtained by annealing the single-stranded DNA oligonucleotides 5′-GATCCGCGGCCGCCCTAGGAGGTAAGCCTATCCCTAACCCTCTCCTCGGTCTCGATTCTACGTAGT-3′ and 5′-CTAGACTACGTAGAATCGAGACCGAGGAGAGGGTTAGGGATAGGCTTACCTCCTAGGGCGGCCGCG-3′), bearing the coding sequence for the V5-tag (underlined) as well as a *Not*I restriction site. In a second step, the PbPL coding sequence and 1067 bp of the upstream region (endogenous promoter) were amplified in two parts from *P*. *berghei* genomic DNA. The upstream region and the N-terminus of PbPL were amplified with primer PbPL-5FR-fw (containing a *Sac*II restriction site) and primer PbPL-nterm-rev. The C-terminal part of PbPL was amplified with primer PbPL-cterm-fw and primer PbPL-cterm-rev (containing a *Not*I restriction site). Next, a *Sna*BI restriction site present within the PbPL coding sequence was used to join both parts together in a 3-way-ligation, for which a *Sac*II/*Not*I digested intermediate vector was used. The resulting vector was linearized with *Sac*II and subsequently blunted, followed by *Not*I digestion. This resulted in release of the joined endogenous promoter region and the PbPL coding sequence, which could be then ligated in frame with the V5 tag into the *Eco*RV/*Not*I-digested pL0017.1.2 vector, thereby replacing the *lisp2* promoter and generating pL0017.1.2-5’FR-PbPL-V5. All primer sequences are listed in S1 Table.

### Generation of PbPL-specific antiserum

His-PbPL_195–312_ was expressed from plasmid parallel-1-His-PbPL_195–312_ in the BL21 (DE3) [pAPlacIQ] *E*. *coli* strain and purified using Ni-NTA agarose (Qiagen). For generation of the His-PbPL antiserum, 50 μg of the purified protein were mixed with one volume of Freund’s adjuvant complete (Sigma) and subcutaneously injected into a Balb/c mouse. After two weeks, the mouse was boosted with the same amount of protein mixed with Freund’s adjuvant incomplete (Sigma), followed by a second boost two weeks later. The immunized mouse was sacrificed, blood was collected and antiserum was obtained after centrifugation of the coagulated blood.

### Expression of PbPL-GFP in *P*. *berghei* parasites

The linearized plasmid pL0043^LS^PbPL-GFP^C^mCherry was transfected into blood stage schizonts of the GIMO_ANKA_ parasite line [44] as described previously [46] and selection of transfected parasites was carried out by adding 5-FC (Abcam) to the drinking water of infected mice [47].

### Immunofluorescence assays

3 x 10^4^ HepG2 cells were seeded on coverslips in 24-well plates and infected the following day with *P*. *berghei* sporozoites. At different time points post-infection, cells were fixed with 4% PFA in PBS for 20 minutes at room temperature, followed by permeabilization with ice-cold methanol. Unspecific binding sites were blocked by incubation in 10% FCS in PBS, followed by incubation with primary antibodies (rabbit anti-GFP (Invitrogen), rat anti-V5 (Invitrogen), mouse anti-PbPL, chicken anti-Exp1 and rat anti-MSP1 (both generated at the Bernhard-Nocht Institute, Hamburg, Germany)) and subsequently with fluorescently labeled secondary antibodies (anti-rabbit AlexaFluor488 (Invitrogen), anti-rat AlexaFluor488 (Invitrogen), anti-chicken Cy5 (Dianova), anti-mouse AlexaFluor488 (Invitrogen)) diluted in 10% FCS in PBS. DNA was visualized by staining with 1 μg/ml DAPI (Sigma). Labeled cells were mounted on microscope slides with Dako Fluorescent Mounting Medium (Dako) and analyzed by confocal point scanning microscopy using a Zeiss LSM5 Duo microscope and a Zeiss Plan-Apochromat 63×/1.4 oil objective. Image processing was performed using ImageJ.

### Generation of selectable marker-free mCherry_*hsp70*_ parasites

The construct pL1694 was used to generate the reporter line mCherry_*hsp70*_ that expresses mCherry under control of the *hsp70* regulatory sequences. This construct was used to target the GIMO_ANKA_ mother line using the ‘gene insertion/marker out’ (GIMO transfection) procedure [44]. To create pL1694, we modified the existing construct pL1628 [44]; this is a Pb*230p* GIMO targeting construct that contains a gene encoding mCherry where expression is driven by the *P*. *berghei eef1α* promoter and transcription is terminated by a *P*. *berghei dhfr* 3’ UTR region. We removed both the *eef1α* promoter and 3’ *dhfr* UTR regions from pL1628 and replaced them with the promoter region and 3’ UTR (transcription terminator) sequences of *P*. *berghei hsp70* (PBANKA_071190). The *hsp70* regulatory regions were amplified from *P*. *berghei* ANKA genomic DNA using primers Hsp70-Promoter-fw/Hsp70-Promoter-rev for the promoter and primers Hsp70-3'UTR-fw/ Hsp70-3'UTR-rev for the 3’UTR. These promoter and 3’UTR elements were cloned into pL1628 vector using the *Asp*718/*Bam*HI and *Spe*I/*Asp*718 restrictions sites respectively. This construct was linearized by digestion with *Ksp*I before transfection. The linearized DNA construct was introduced into GIMO_ANKA_ parasites using standard methods of GIMO-transfection [44]. Transfected parasites were selected in mice by applying negative selection by providing 5-FC in the drinking water of mice [47]. Negative selection results in selection of parasites where the *hdhfr*::*yfcu* selectable marker in the *230p* locus is replaced by the mCherry reporter-cassette. Selected transgenic parasites (mCherry_*hsp70*_) were cloned by limiting dilution. Correct integration of the constructs into the genome of mCherry_*hsp70*_ parasites was analyzed by diagnostic PCR on parasite gDNA. All primer sequences are listed in S1 Table.

### Generation of PbPL-KO parasites and PbPL complementation

PbPL-KO parasites were generated by transfection of the plasmoGEM vector PbGEM-099883 [27,28] into mCherry_*hsp70*_ parasites as described before [46], targeting the PbPL coding sequence by double crossover homologous recombination, and were selected by pyrimethamine (Sigma). For generation of marker-free KO parasites, the selectable marker was removed by negative selection with 5-FC in the drinking water of infected mice as described previously [47]. Subsequently, complemented parasites were generated by transfection of the linearized pL0017.1.2-5’FR-PbPL-V5 vector into marker-free KO parasites, leading to expression of PbPL-V5 under its endogenous promoter from the *c*- or *d-ssu-rRNA* locus. Parasite genomic DNA (gDNA) was isolated from 0.05% saponin-treated infected red blood cells using the Nucleospin Blood QuickPure kit (Macherey-Nagel) and all genetic modifications of parasites were confirmed by diagnostic PCR using GoTaq Flexi DNA polymerase. All primer sequences are listed in S1 Table. Clonal parasite lines were either generated by detached cell injection [48] or by limiting dilution.

### Analysis of mosquito stage development

Exflagellation of male gametocytes was analyzed using standard *in vitro* gametocyte activation assays basically as described previously [49]. In brief, 2 μl of tail blood were added to 8 μl of ookinete medium (RPMI1640 containing 25 mM HEPES, 20% FCS, 100 μM xanthurenic acid [pH 7.4]) and the mixture was placed under a Vaseline-coated coverslip. By light microscopy (100x objective) we counted the number of unemerged but activated male gametocytes (defined as activated male gametocytes with moving flagella inside the erythrocyte) and of emerged activated male gametocytes (with extracellular male gametes) in four 2 minute intervals starting at 8 minutes post-activation. 20 minutes post-activation, the characteristic exflagellation centers were counted in 20 fields of view using a light microscope (40x objective).

9 days after the infective blood meal, midguts of 15–23 mosquitoes were dissected into PBS and the pooled midguts were fixed in 4% paraformaldehyde (PFA) in PBS for 20 minutes at room temperature. The midguts were then washed with PBS and stored in PBS at 4°C in the dark. The next day, fixed midguts were mounted on glass slides containing a small amount of Dako Fluorescent Mounting Medium and imaged using a fluorescence microscope with a 5x objective. The average number of oocysts per midgut was then determined using an ImageJ-based counting macro [50].

On day 18 and 26 after the infective blood meal, mosquito midguts and salivary glands were harvested for determination of sporozoite numbers. 10 mosquitoes were dissected, organs pooled and homogenized, and released sporozoites were counted using a hemocytometer. For determination of hemolymph sporozoite numbers, hemolymph from 10 mosquitoes was collected on day 18 after the infective blood meal by perfusion of the thorax and abdomen with 50 μl of PBS and sporozoites were counted using a hemocytometer.

### *In vivo* analysis of PbPL-KO parasites

1,000 WT, KO or complemented sporozoites were injected intravenously into 6–7 C57BL/6 mice per group. Subsequently, blood stage parasitemia was determined between day 3 and 6 post-infection by FACS analysis using a FACSCalibur flow cytometer (BD Biosciences) and the mCherry fluorescence of parasites.

For determination of blood stage growth in mice, 1,000 infected red blood cells (containing mixed blood stages) of WT, KO or complemented parasites were injected intravenously into 4–5 C57BL/6 mice per group and subsequent parasitemia was determined between day 3 and 6 post-infection by FACS analysis.

For determination of liver loads, 4–5 C57BL/6 mice per group were injected intravenously with 10,000 WT, KO or complemented sporozoites. After 38 hours, whole livers were removed and homogenized on ice in 5 ml of denaturing solution (4 M guanidine thiocyanate, 25 mM sodium citrate [pH 7.0], 0.5% N-lauroylsarcosine, 0.7% β-mercaptoethanol) using a Polytron homogenizer (Kinematica). Total RNA was isolated from 100 μl of liver homogenate using 900 μl of TRIzol (Ambion). Random-primed cDNA synthesis was performed using 2 μg of total RNA and GoScript reverse transcriptase. Levels of parasite 18S ribosomal RNA and mouse hypoxanthine guanine phosphoribosyltransferase (HPRT) cDNAs obtained from the reaction were quantified by real-time PCR using previously described primers [10]: Pb18S-fw and Pb18S-rev for *P*. *berghei* 18S ribosomal RNA and MmHPRT-fw and MmHPRT-rev for the *Mus musculus* housekeeping gene *hprt*. To quantify gene expression, MESA GREEN qPCR MasterMix Plus (Eurogentec) was used according to the manufacturer's instructions. Reactions were performed in triplicate in an ABI Prism 7000 sequence Detection System (Applied Biosystems) with 1 μl of cDNA in a total volume of 25 μl and the following reaction conditions: 1 step of 2 min at 50°C, 1 step of 5 min at 95°C, 40 cycles of 15 sec at 95°C and 1 min at 60°C. Relative expression levels were calculated using the ΔΔCt method [51]. All primer sequences are listed in S1 Table.

### Determination of sporozoite infectivity *in vitro*

5 x 10^4^ HepG2 cells per well were seeded in 24-well plates and infected the next day with 10,000 WT, KO and complemented sporozoites. After 48 hpi, the average number of infected host cells per well was quantified in triplicate.

### Parasite size measurement and detached cell analysis

5 x 10^4^ HepG2 cells per well were seeded in 24-well plates and infected the next day with WT, KO and complemented sporozoites. 48 hpi, parasite size (area) was determined by density slicing using ImageJ and infected cells were counted. At 65 hpi, the number of detached cells (DCs) in the supernatant was counted in triplicate. The percentage of DC formation was then calculated by dividing the number of DCs in the supernatant by the number of infected cells at 48 hpi. For quantification of DC morphology, DCs were harvested at 65 hpi and DCs with normal (merozoites freely distributed in host cell cytoplasm) and abnormal morphology (merozoites still being clustered) were counted.

### Stage quantification assay

3 x 10^4^ HepG2 cells per well were seeded in 24-well plates on coverslips and infected the next day with WT, KO and complemented sporozoites. They were fixed at 54 and 65 hpi and stained for IFA with an anti-MSP1 antiserum as already described. Subsequently, attached hepatocytes containing schizont, cytomere and merozoite stage parasites were counted based on parasite plasma membrane morphology.

### PVM rupture assay

2 × 10^6^ HepG2 cells were transfected with 4 μg pEGFP-N3 plasmid (Clontech) using an Amaxa nucleofector (Lonza) as described previously [30] and subsequently seeded into 4-chamber glass bottom dishes (In Vitro Scientific). The following day, two wells of GFP-expressing cells were infected with mCherry-expressing WT and the other two either with KO or complemented sporozoites. The percentage of merozoite-forming parasites that rupture the PVM, as defined by influx of GFP into the PV, as well as the time between successful formation of merozoites and PVM rupture was subsequently analyzed by live-cell time-lapse imaging. For this, a Zeiss LSM5 Duo microscope with a Zeiss Plan-Apochromat 63×/1.4 oil objective was used in the LIVE mode (confocal line scanning). Development of parasites shortly before or in the cytomere stage was followed for 12 hours starting between 54 and 56 hpi using the Zeiss LSM Multitime-Macro and an image was acquired every 10 minutes. During imaging, cells were kept in a CO_2_ incubator at 37°C. Only parasites that developed to the merozoite stage within the first 6 hours of imaging and displayed completely normal development (e.g. absence of merofusosomes [52]) were used for further analysis. Image processing was performed using ImageJ.

### Statistical analyses

Statistical analyses were performed using GraphPad Prism (GraphPad Software). For comparisons between groups, a one-way analysis of variance (ANOVA) followed by a Holm-Sidak multiple comparison test was perfomed. P values of < 0.05 were considered significant.

### Accession numbers

*P*. *berghei* phospholipase (PBANKA_112810), *P*. *berghei* glyceraldehyde-3-phosphate dehydrogenase (PBANKA_132640), *P*. *berghei* LISP2 (PBANKA_100300), *P*. *berghei* exported protein 1 (PBANKA_092670), *P*. *berghei* heat shock protein 70 (PBANKA_071190), *P*. *berghei* 18S ribosomal RNA (berg07_18S), P. *berghei* merozoite surface protein 1 (PBANKA_083100), *M*. *musculus* hypoxanthine guanine phosphoribosyltransferase (NM_013556.2).

## Supporting Information

S1 Fig Generation of the marker-free *P*. *berghei* ANKA reporter line mCherry_*hsp70*_, expressing mCherry under the control of the *hsp70* regulatory sequences. A) Schematic representation showing the introduction of the mCherry-reporter cassette (pL1694) into the GIMO_ANKA_ parasite line. Construct pL1694 contains the *hsp70* promoter (*5’hsp70*)-*mCherry-3’hsp70* (terminator) cassette. The construct integrates into the modified *P*. *berghei 230p* locus containing the *hdhfr*::*yfcu* selectable marker cassette by double crossover homologous recombination at the target regions (grey boxes). Negative selection with 5-Fluorocytosine selects for the transgenic parasite line mCherry_*hsp70*_ that has the mCherry-reporter cassette introduced into the *230p* locus and the *hdhfr*::*yfcu* marker removed. Location of primers used for PCR analysis are shown. B) Diagnostic PCR-analysis confirms the correct integration of construct pL1694 into the *P*. *berghei* genome. Diagnostic PCR-analysis shows the absence of the *hdhfr*::*yfcu* marker and the correct integration of the mCherry expression cassette into the mCherry_*hsp70*_ genome. In case of successful integration, primers 1 and 2 are expected to yield a PCR product of 951 bp and primers 3 and 4 a product of 1056 bp. Primers 5 and 6 amplify mCherry and give a product of 718 bp, while 7 and 8 bind within the selectable marker resulting in a product of 1108 bp. C) mCherry expression in midgut (MG) oocysts and salivary gland (SG) sporozoites of mosquitoes 20 days after infection with mCherry_*hsp70*_. D) Oocyst of mCherry_*hsp70*_ parasites 12 days after infection. E) Individual salivary gland sporozoites of mCherry_*hsp70*_ parasites 18 days after infection. Scale bars = 10 μm. All primer sequences are listed in S1 Table.
(TIFF)

S2 Fig Mosquito stage development of PbPL-knockout parasites. A, B) Male gametocytes of PbPL-knockout (KO) parasites emerge normally in time and numbers. The proportion of exflagellating male gametocytes of wild-type (WT), PbPL-KO (KO2) and complemented PbPL-KO (CMP2) that had emerged from their host erythrocyte was scored by light microscopy at different times after the induction of gametogenesis *in vitro* (A). 20 minutes after induction, the average number of exflagellation centers per field of view was determined using a 40x objective (B). C) PbPL-KO parasites produce normal numbers of oocysts. 9 days after the infective blood meal, midguts were removed and for each parasite line the average number of oocysts per midgut was determined from 15–23 mosquitoes per experiment. D, E, F) PbPL-KO sporozoites have a defect in egress from oocysts. 18 and 26 days after the infective blood meal, the average number of sporozoites in the mosquito midgut (D) or salivary glands (F) was quantified. In addition, the average number of sporozoites in the hemolymph was determined 18 days after the infective blood meal (E). For each mosquito feed, 10 mosquitoes were dissected and sporozoites were counted. For all experiments means +/− SD of 4–8 independent mosquito feed experiments are shown. For statistical analysis a one-way ANOVA followed by a Holm-Sidak multiple comparison test was performed. All statistically significant differences are indicated by asterisks (* p < 0.05, ** p < 0.01, *** p < 0.001, **** p < 0.0001).
(TIFF)

S3 Fig PbPL-knockout parasites show normal blood stage development and liver infection levels *in vivo* (related to Fig. 4). A) The blood stage multiplication rate of PbPL-knockout (KO2) parasites does not differ from wild-type (WT) and complemented PbPL-KO (CMP2) parasites. The blood stage multiplication rate was calculated by dividing the parasitemia determined by FACS analysis (Fig. 4) of each individual mouse at day 5 and day 6 after sporozoite injection by the parasitemia the respective mouse had one day before. Shown are means +/− SD of 6–7 mice per group. B) Blood stage growth curve of WT, KO2 and CMP2 parasites. 1,000 mixed blood stage parasites were injected intravenously into C57BL/6 mice and subsequent parasitemia was measured by FACS analysis. Shown are means +/− SD of 8–9 mice per group obtained in two independent experiments. C) PbPL-KO parasites show similar liver loads in comparison to WT and CMP2 parasites. C57BL/6 mice were injected intravenously with 10,000 WT, KO2 or CMP2 sporozoites. After 38 hours, total RNA was isolated from whole infected livers and levels of 18S ribosomal parasite RNA (Pb18S) and mouse hypoxanthine guanine phosphoribosyltransferase (MmHPRT) mRNA were quantified by real-time PCR. Relative amounts of parasite 18S ribosomal RNA were normalized against the expression levels of mouse HPRT and infection levels of WT parasites were set to 100%. Shown are means +/− SD of 4–5 mice per group. There was no statistically significant difference in liver infection levels between the groups (one-way ANOVA, p = 0.5567).
(TIFF)

S4 Fig PbPL does not affect liver stage growth but plays a role in detached cell formation (related to Fig. 5). A) Both clonal PbPL-knockout (KO) parasite lines develop normally in size. HepG2 cells were infected with wild-type (WT) and PbPL-KO (KO1 and KO2) sporozoites. 48 hpi, parasite size (area) was determined by density slicing using ImageJ. For each parasite line, the average size of 50–100 parasites was determined in each experiment. B) PbPL-KO parasites show normal MSP1 and ExpI expression and localization. HepG2 cells were infected with WT and KO2 sporozoites, fixed at 60 hpi and analyzed by IFA using an antiserum against the plasma membrane marker protein MSP1 (green) and the PVM marker protein ExpI (purple). The merged channels additionally contain DAPI-stained nuclei (blue). Scale bars = 10 μm. C) Both clonal PbPL-KO parasite lines produce fewer detached cells (DCs). DCs in the supernatant were counted at 65 hpi in triplicate and were normalized to the number of infected cells at 48 hpi. For all experiments means +/− SD of three independent experiments are shown. For statistical analysis, a one-way ANOVA followed by a Holm-Sidak multiple comparison test was performed (** p < 0.01, n.s. = not significant). Parasite sizes in (A) did not differ statistically significantly from each other (one-way ANOVA, p = 0.9531).
(TIFF)

S1 Movie Successful PVM rupture of a wild-type parasite (related to Fig. 7). HepG2 cells expressing GFP (green) were infected with mCherry-expressing wild-type sporozoites (red). Parasite development was followed by confocal live-cell time-lapse microscopy and imaging was started around 55 hpi. 4 frames per second and 10 minutes time interval between each frame. Scale bar = 10 μm.
(AVI)

S2 Movie Unsuccessful PVM rupture of a PbPL-knockout parasite (related to Fig. 7). HepG2 cells expressing GFP (green) were infected with mCherry-expressing PbPL-knockout (KO2) sporozoites (red). Parasite development was followed by confocal live-cell time-lapse microscopy and imaging was started around 55 hpi. 4 frames per second and 10 minutes time interval between each frame. Scale bar = 10 μm.
(AVI)

S3 Movie Successful PVM rupture of a complemented PbPL-knockout parasite (related to Fig. 7). HepG2 cells expressing GFP (green) were infected with mCherry-expressing complemented PbPL-knockout (CMP2) sporozoites (red). Parasite development was followed by confocal live-cell time-lapse microscopy and imaging was started around 55 hpi. 4 frames per second and 10 minutes time interval between each frame. Scale bar = 10 μm.
(AVI)

S1 Table Primers used in this study. Restriction sites are underlined if present.
(PDF)
