# Supplementary figures and images for: A Plasmodium Phospholipase Is Involved in Disruption of the Liver Stage Parasitophorous Vacuole Membrane
Source: PLoS Pathog. 2015 Mar 18;11(3):e1004760. doi: 10.1371/journal.ppat.1004760 (PMC4364735; doi:10.1371/journal.ppat.1004760)

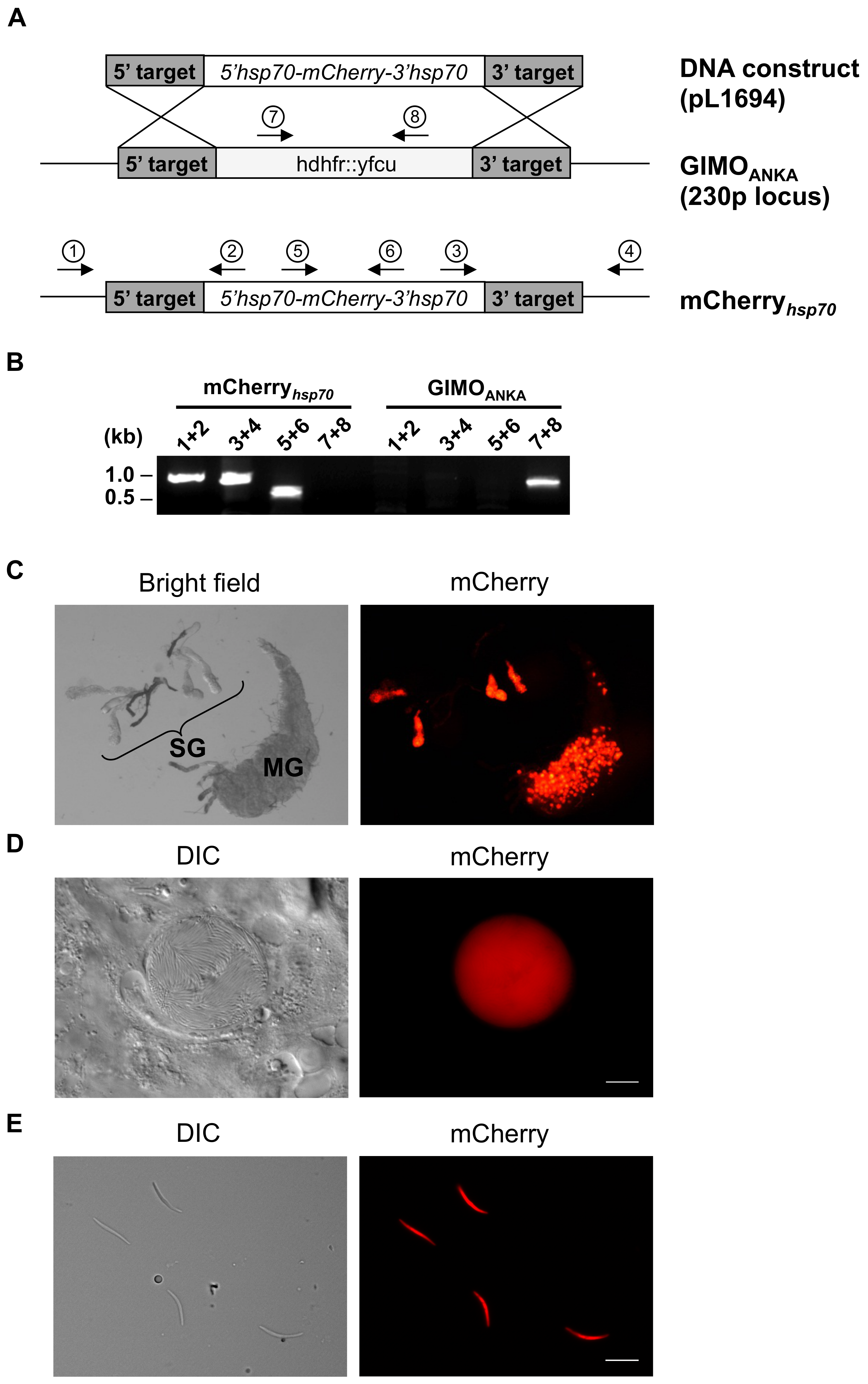

Supplement: S1 Fig — A) Schematic representation showing the introduction of the mCherry-reporter cassette (pL1694) into the GIMOANKA parasite line. Construct pL1694 contains the hsp70 promoter (5’hsp70)-mCherry-3’hsp70 (terminator) cassette. The construct integrates into the modified P. berghei 230p locus containing the hdhfr::yfcu selectable marker cassette by double crossover homologous recombination at the target regions (grey boxes). Negative selection with 5-Fluorocytosine selects for the transgenic parasite line mCherryhsp70 that has the mCherry-reporter cassette introduced into the 230p locus and the hdhfr::yfcu marker removed. Location of primers used for PCR analysis are shown. B) Diagnostic PCR-analysis confirms the correct integration of construct pL1694 into the P. berghei genome. Diagnostic PCR-analysis shows the absence of the hdhfr::yfcu marker and the correct integration of the mCherry expression cassette into the mCherryhsp70 genome. In case of successful integration, primers 1 and 2 are expected to yield a PCR product of 951 bp and primers 3 and 4 a product of 1056 bp. Primers 5 and 6 amplify mCherry and give a product of 718 bp, while 7 and 8 bind within the selectable marker resulting in a product of 1108 bp. C) mCherry expression in midgut (MG) oocysts and salivary gland (SG) sporozoites of mosquitoes 20 days after infection with mCherryhsp70. D) Oocyst of mCherryhsp70 parasites 12 days after infection. E) Individual salivary gland sporozoites of mCherryhsp70 parasites 18 days after infection. Scale bars = 10 μm. All primer sequences are listed in S1 Table. (TIFF) [file ppat.1004760.s001.tiff]

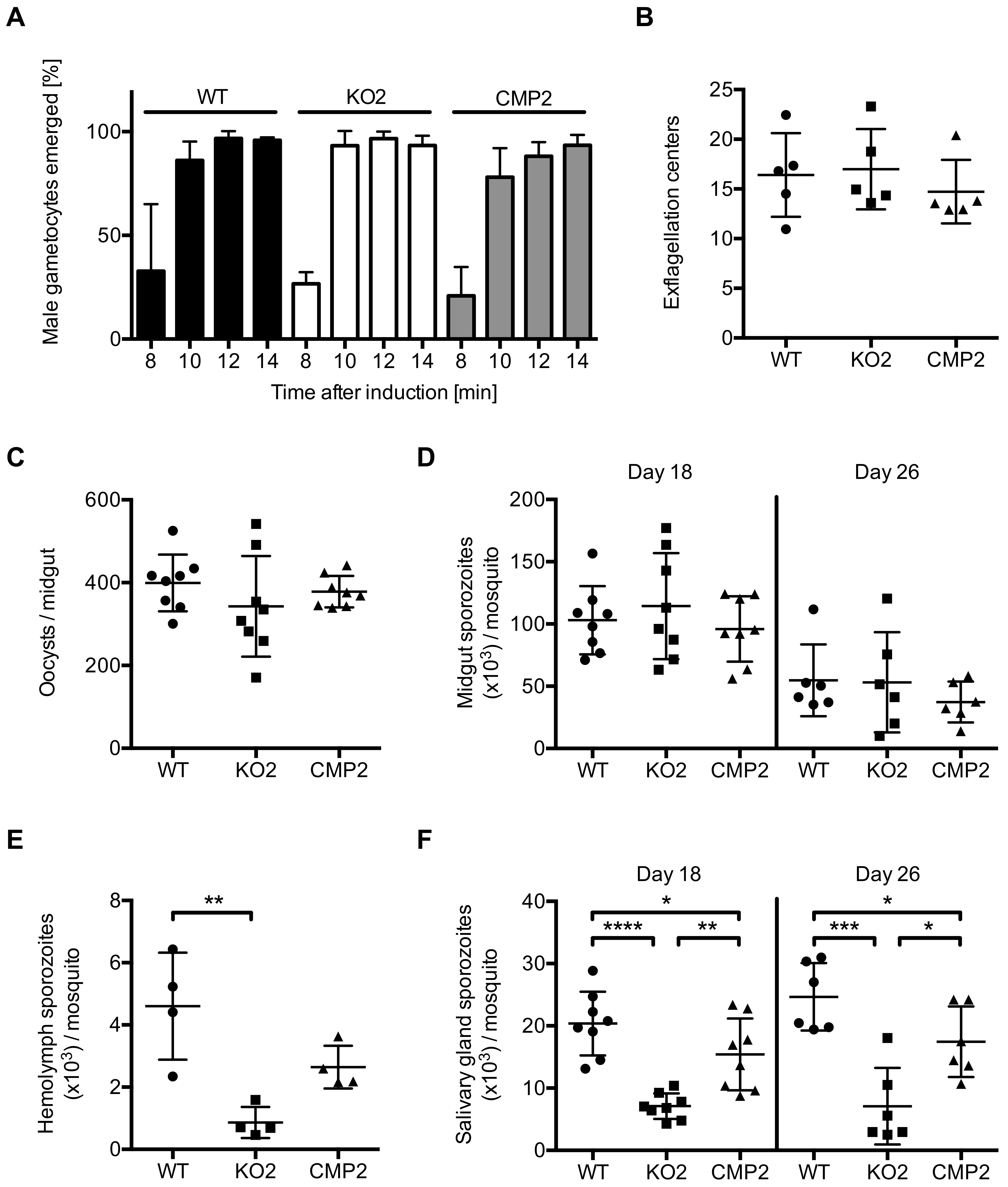

Supplement: S2 Fig — A, B) Male gametocytes of PbPL-knockout (KO) parasites emerge normally in time and numbers. The proportion of exflagellating male gametocytes of wild-type (WT), PbPL-KO (KO2) and complemented PbPL-KO (CMP2) that had emerged from their host erythrocyte was scored by light microscopy at different times after the induction of gametogenesis in vitro (A). 20 minutes after induction, the average number of exflagellation centers per field of view was determined using a 40x objective (B). C) PbPL-KO parasites produce normal numbers of oocysts. 9 days after the infective blood meal, midguts were removed and for each parasite line the average number of oocysts per midgut was determined from 15–23 mosquitoes per experiment. D, E, F) PbPL-KO sporozoites have a defect in egress from oocysts. 18 and 26 days after the infective blood meal, the average number of sporozoites in the mosquito midgut (D) or salivary glands (F) was quantified. In addition, the average number of sporozoites in the hemolymph was determined 18 days after the infective blood meal (E). For each mosquito feed, 10 mosquitoes were dissected and sporozoites were counted. For all experiments means +/− SD of 4–8 independent mosquito feed experiments are shown. For statistical analysis a one-way ANOVA followed by a Holm-Sidak multiple comparison test was performed. All statistically significant differences are indicated by asterisks (* p < 0.05, ** p < 0.01, *** p < 0.001, **** p < 0.0001). (TIFF) [file ppat.1004760.s002.tiff]

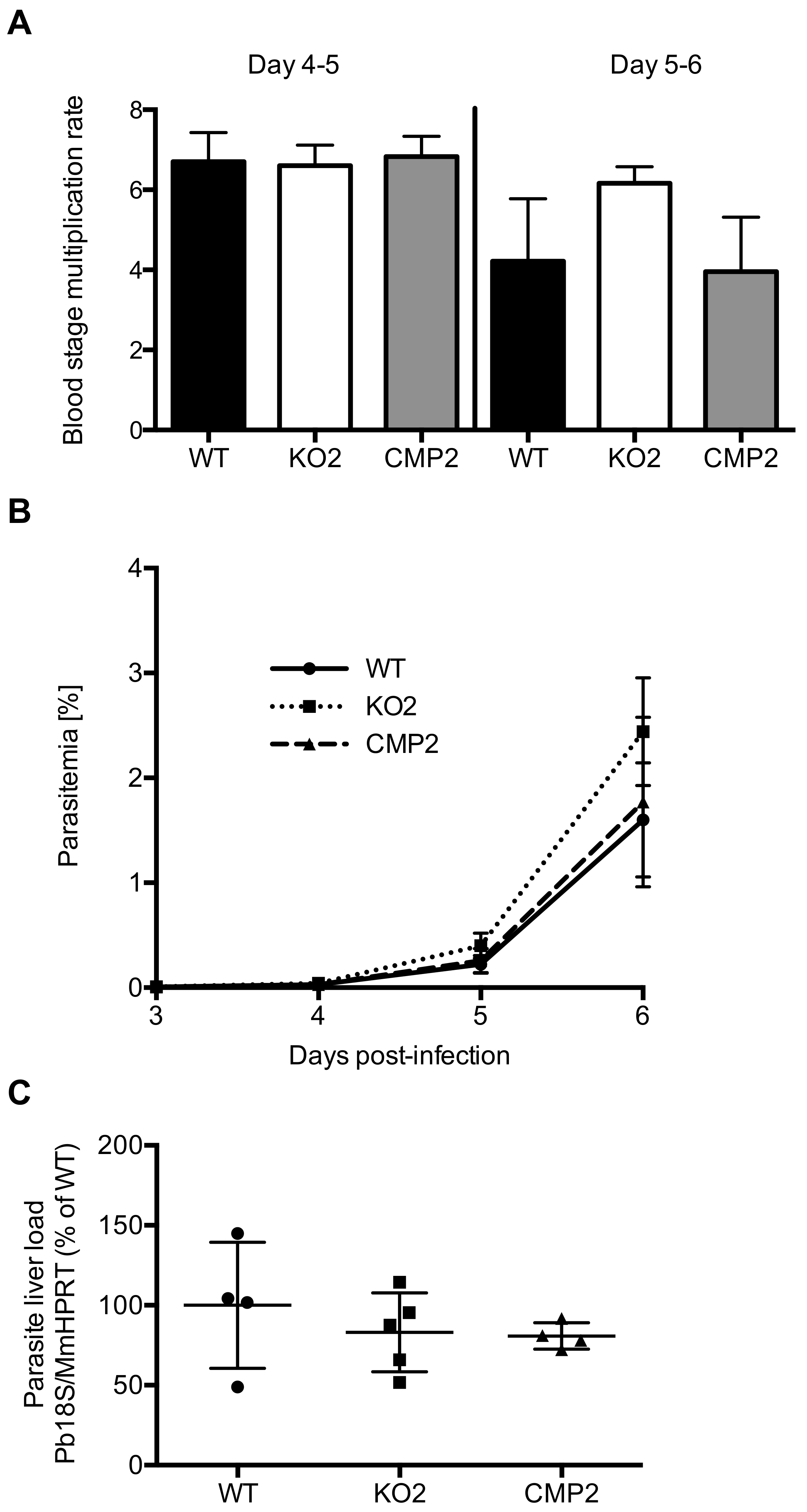

Supplement: S3 Fig — A) The blood stage multiplication rate of PbPL-knockout (KO2) parasites does not differ from wild-type (WT) and complemented PbPL-KO (CMP2) parasites. The blood stage multiplication rate was calculated by dividing the parasitemia determined by FACS analysis (Fig. 4) of each individual mouse at day 5 and day 6 after sporozoite injection by the parasitemia the respective mouse had one day before. Shown are means +/− SD of 6–7 mice per group. B) Blood stage growth curve of WT, KO2 and CMP2 parasites. 1,000 mixed blood stage parasites were injected intravenously into C57BL/6 mice and subsequent parasitemia was measured by FACS analysis. Shown are means +/− SD of 8–9 mice per group obtained in two independent experiments. C) PbPL-KO parasites show similar liver loads in comparison to WT and CMP2 parasites. C57BL/6 mice were injected intravenously with 10,000 WT, KO2 or CMP2 sporozoites. After 38 hours, total RNA was isolated from whole infected livers and levels of 18S ribosomal parasite RNA (Pb18S) and mouse hypoxanthine guanine phosphoribosyltransferase (MmHPRT) mRNA were quantified by real-time PCR. Relative amounts of parasite 18S ribosomal RNA were normalized against the expression levels of mouse HPRT and infection levels of WT parasites were set to 100%. Shown are means +/− SD of 4–5 mice per group. There was no statistically significant difference in liver infection levels between the groups (one-way ANOVA, p = 0.5567). (TIFF) [file ppat.1004760.s003.tiff]

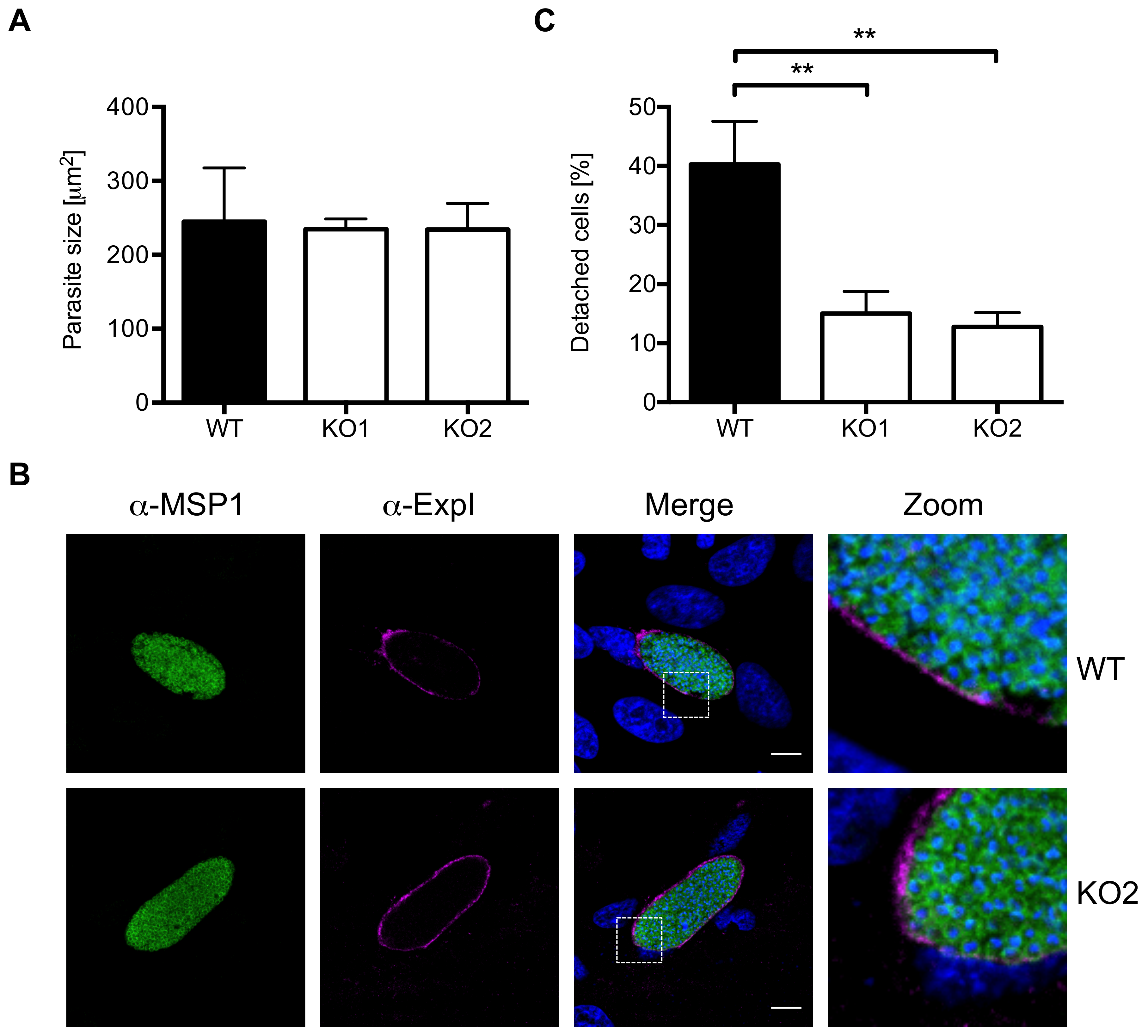

Supplement: S4 Fig — A) Both clonal PbPL-knockout (KO) parasite lines develop normally in size. HepG2 cells were infected with wild-type (WT) and PbPL-KO (KO1 and KO2) sporozoites. 48 hpi, parasite size (area) was determined by density slicing using ImageJ. For each parasite line, the average size of 50–100 parasites was determined in each experiment. B) PbPL-KO parasites show normal MSP1 and ExpI expression and localization. HepG2 cells were infected with WT and KO2 sporozoites, fixed at 60 hpi and analyzed by IFA using an antiserum against the plasma membrane marker protein MSP1 (green) and the PVM marker protein ExpI (purple). The merged channels additionally contain DAPI-stained nuclei (blue). Scale bars = 10 μm. C) Both clonal PbPL-KO parasite lines produce fewer detached cells (DCs). DCs in the supernatant were counted at 65 hpi in triplicate and were normalized to the number of infected cells at 48 hpi. For all experiments means +/− SD of three independent experiments are shown. For statistical analysis, a one-way ANOVA followed by a Holm-Sidak multiple comparison test was performed (** p < 0.01, n.s. = not significant). Parasite sizes in (A) did not differ statistically significantly from each other (one-way ANOVA, p = 0.9531). (TIFF) [file ppat.1004760.s004.tiff]
