# Supplementary material for: A Plasmodium Phospholipase Is Involved in Disruption of the Liver Stage Parasitophorous Vacuole Membrane
Source: PLoS Pathog. 2015 Mar 18;11(3):e1004760. doi: 10.1371/journal.ppat.1004760 (PMC4364735; doi:10.1371/journal.ppat.1004760)
Supplement: S1 Table — Restriction sites are underlined if present. (PDF) [file ppat.1004760.s008.pdf]

Name	Lab-No.	Sequence (5’-3’)	Purpose
PbPL-expression-fw	166	GAATTAAAGAAGTCACAGCCGA	PbPL gene expression analysis (Figure 1)
PbPL-expression-rev	167	CTTTCTCCGTTTCACTAAGCA	PbPL gene expression analysis (Figure 1)
GAPDH-expression-fw	180	GAAGTGACATTGAAGTAGTTGC	GAPDH gene expression analysis (Figure 1)
GAPDH-expression-rev	181	TAATTGGTTGGCTGTTGAGG	GAPDH gene expression analysis (Figure 1)
PbPL-antiserum-fw	361	GCGGATCCTAAGGAAAAAGAATACGTATCAGAGCATAC	His-$PbPL_{195-312}$ expression in bacteria for generation of antiserum (Figure 1)
PbPL-antiserum-rev	362	GCCTCGAGCTAAATTTCCATACCATCGTCAACAGTACC	His-$PbPL_{195-312}$ expression in bacteria for generation of antiserum (Figure 1)
PbPL-GFP-fw	286	GCAGATCTATGCAAGCATTTCTCTTTATAATC	PbPL-GFP expression in liver stage parasites (Figure 1)
PbPL-GFP-rev	287	GCAGATCTATTAGTTGTATTTTTATCTATGATATTG	PbPL-GFP expression in liver stage parasites (Figure 1)
mCherry-fw	41	CGTAGGTACCAGCTTAATTCTTTTCGAGCTCTTT	PbPL-GFP expression in liver stage parasites (Figure 1)
mCherry-rev	42	ACTGGGTACCCGAAATTGAAGGAAAAAACATCATTTG	PbPL-GFP expression in liver stage parasites (Figure 1)
Hsp70-Promoter-fw	6254	CCGGGGTACCGTAATATTTTGTTGGTGAGC	Generation of $mCherry_{hsp70}$ parasites (Figure S1)
Hsp70-Promoter-rev	4213	GGGGGATCCCTTTTTTTAATTGTAATTGTAATTTATTGG	Generation of $mCherry_{hsp70}$ parasites (Figure S1)
Hsp70-3'UTR-fw	4214	GGGACTAGTTATTATTGTTCTGTACTTCTTTTGTG	Generation of $mCherry_{hsp70}$ parasites (Figure S1)
Hsp70-3'UTR-rev	4215	GGGGGTACCGGAAAATACCAATAATACCG	Generation of $mCherry_{hsp70}$ parasites (Figure S1)
Primer-1	5510	GCAAAGTGAAGTTCAAATATGTG	Diagnostic PCR of $GIMO_{ANKA}$ and $mCherry_{hsp70}$ parasites (Figure S1)
Primer-2	6836	CGCAATTTGTTGTACATAAAATAG	Diagnostic PCR of $GIMO_{ANKA}$ and $mCherry_{hsp70}$ parasites (Figure S1)
Primer-3	6841	AAAATAAAAGGAGGGATCTAGAG	Diagnostic PCR of $GIMO_{ANKA}$ and $mCherry_{hsp70}$ parasites (Figure S1)
Primer-4	5511	AGTGACTTTCAGTGAAATCGC	Diagnostic PCR of $GIMO_{ANKA}$ and $mCherry_{hsp70}$ parasites (Figure S1)
Primer-5	6663	GTGAGCAAGGGCGAGGAG	Diagnostic PCR of $GIMO_{ANKA}$ and $mCherry_{hsp70}$ parasites (Figure S1)
Primer-6	5514	CTTGTACAGCTCGTCCATGC	Diagnostic PCR of $GIMO_{ANKA}$ and $mCherry_{hsp70}$ parasites (Figure S1)
Primer-7	4698	GTTCGCTAAACTGCATCGTC	Diagnostic PCR of $GIMO_{ANKA}$ and $mCherry_{hsp70}$ parasites (Figure S1)
Primer-8	4699	GTTTGAGGTAGCAAGTAGACG	Diagnostic PCR of $GIMO_{ANKA}$ and $mCherry_{hsp70}$ parasites (Figure S1)
Primer-1	424	TGCTCTTCTACCAAGATACGCACT	Diagnostic PCR of WT, KO and KO-MF parasites (Figure 2)

Primer-2	425	TGCATACCCAAATGGCGAAT	Diagnostic PCR of WT, KO and KO-MF parasites (Figure 2)
Primer-3	420	CTTTGGTGACAGATACTAC	Diagnostic PCR of WT, KO and KO-MF parasites (Figure 2)
Primer-4	426	CCCAAAGGTGCTGAATATGCATTCCGT	Diagnostic PCR of WT, KO and KO-MF parasites (Figure 2)
Primer-5	577	GTGACAGGGGGAATG	Diagnostic PCR of WT, KO and KO-MF parasites (Figure 2)
Primer-6	578	GATAGCACTACCACCGG	Diagnostic PCR of WT, KO and KO-MF parasites (Figure 2)
Primer-7	579	CCACCAATTTAAAGATAGTGTG	Diagnostic PCR of WT, KO and KO-MF parasites (Figure 2)
PbPL-5FR-fw	646	AACCGCGGCGGAAAATTTCATTGTATTACATCAAT	Generation of complemented KO parasites (Figure 3)
PbPL-nterm-rev	637	TCTTCTGTATGTTCTGATATGG	Generation of complemented KO parasites (Figure 3)
PbPL-cterm-fw	579	CCACCAATTTAAAGATAGTGTG	Generation of complemented KO parasites (Figure 3)
PbPL-cterm-rev	632	AAGCGGCCGCATTAGTTGTATTTTTATCTATGATATTG	Generation of complemented KO parasites (Figure 3)
Primer-1	728	GTGTAGTAACATCAGTTATTGTGTG	Diagnostic PCR of complemented KO parasites (Figure 3)
Primer-2	729	ATACTGTATAACAGGTAAGCTGTTATTGTG	Diagnostic PCR of complemented KO parasites (Figure 3)
Primer-3	730	TTTCCCAGTCACGACGTTG	Diagnostic PCR of complemented KO parasites (Figure 3)
Primer-4	731	CTTAGTGTTTTGTATTAATGTCGATTTG	Diagnostic PCR of complemented KO parasites (Figure 3)
Pb18S-fw	793	AAGCATTAAATAAAGCGAATACATCCTTAC	Real-time PCR for determination of liver load (Figure S3)
Pb18S-rev	794	GGAGATTGGTTTTGACGTTTATGTG	Real-time PCR for determination of liver load (Figure S3)
MmHPRT-fw	795	GTAATGATCAGTCAACGGGGGAC	Real-time PCR for determination of liver load (Figure S3)
MmHPRT-rev	796	CCAGCAAGCTTGCAACCTTAACCA	Real-time PCR for determination of liver load (Figure S3)
